# Metabolic syndrome and liver fibrosis in nondiabetic MASLD: A cross-sectional double machine learning study

**DOI:** 10.1097/MD.0000000000051316

**Published:** 2026-11-06

**Authors:** Van Trung Huynh, Lua Thi Ngo, Hien-Huu Tran

**Affiliations:** a Center for Gastrointestinal Endoscopy and Endoscopic Surgery, Tam Anh General Hospital, Ho Chi Minh City, Vietnam; b School of Biomedical Engineering, International University, Vietnam National University Ho Chi Minh City, Ho Chi Minh City, Vietnam; c Thien Nha 399 Hepatology Clinic, Ho Chi Minh City, Vietnam.

**Keywords:** causal inference, double machine learning, FIB-4, hypothesis-generating study, liver fibrosis, metabolic dysfunction–associated steatotic liver disease, metabolic syndrome, NHANES, prediabetes, transient elastography

## Abstract

Metabolic syndrome (MetS) is associated with hepatic fibrosis in metabolic dysfunction–associated steatotic liver disease (MASLD), but whether this association is causal in nondiabetic MASLD has not been assessed with semiparametric causal methods. We analyzed 1306 nondiabetic adults with MASLD from the National Health and Nutrition Examination Survey 2017 to 2020; 959 (73.4%) had prediabetes. The contrast was the supra-threshold MetS phenotype (≥3/5 harmonized components) versus the subthreshold phenotype (0–2/5), estimated as a covariate-standardized stratum contrast. Three co-primary outcomes were prespecified: fibrosis-4 index (FIB-4) ≥ 1.3, age-residualized FIB-4, and age-independent FibroScan ≥ 7.9 kPa. We applied double machine learning, augmented inverse probability weighting, causal forests, and 4 meta-learners, with 20 robustness analyses prespecified and 9 added at peer review. Estimates diverged with the age dependence of the outcome. For FIB-4 the adjusted risk difference was 0.153 (95% confidence interval [CI], 0.057–0.250; *P* = .002; *E* value = 3.75), or 0.095 (0.050–0.140) under a constant-effect specification; across 10 specifications estimates ranged from 0.078 to 0.163, attenuating to 0.091 (95% CI, −0.018 to 0.201; *P* = .10) after age residualization and to 0.001 (95% CI, −0.094 to 0.097; *P* = .98) for FibroScan, whose interval excludes the FIB-4 estimate at equal precision. Age stratification showed no effect below 40, with heterogeneity for FIB-4 (*I*^2^ = 72%) and age-residualized FIB-4 (*I*^2^ = 73%) but none for FibroScan (*I*^2^ = 0%), so the gradient is specific to FIB-4. Shapley decomposition ranked fasting glucose and central obesity highest, but every component’s CI included zero, and no component pair showed additive-scale interaction after adjustment. Selection into the MASLD sample was exposure-dependent (40.7% vs 30.7%; *P* < .001); weighting gave 0.126 (0.003–0.250). The supra-threshold MetS phenotype was associated with a higher prevalence of FIB-4–defined fibrosis risk but not with age-independent elastography. The divergence tracks the outcome measure rather than sample composition: perturbations addressing what FIB-4 measures converge on 0.08 to 0.10, while those addressing sample composition leave it near 0.13 to 0.15. These findings generate hypotheses for confirmatory longitudinal testing rather than definitive causal evidence. They apply to the predominantly prediabetic MASLD population studied here and cannot be extrapolated to metabolically healthy or lean MASLD.

## 1. Introduction

Metabolic dysfunction–associated steatotic liver disease (MASLD), formerly nonalcoholic fatty liver disease (NAFLD),^[1]^ affects approximately 30% of the global adult population, with a prevalence rising to 38% in the most recent estimates.^[2]^ Although cardiovascular disease remains the leading cause of mortality in MASLD, hepatic fibrosis stage is the strongest predictor of liver-related outcomes: a systematic review and meta-analysis found that patients with advanced fibrosis (≥F3) face a 3-fold higher risk of all-cause mortality and a 10-fold higher risk of liver-related mortality compared with those without fibrosis,^[3]^ a gradient also observed in prospective cohort follow-up.^[4]^ Identifying modifiable causes of fibrosis progression is a clinical priority.

Metabolic syndrome (MetS), defined as the co-occurrence of central obesity, dyslipidemia, hypertension, and dysglycemia,^[5]^ is present in more than half of MASLD patients and has been consistently associated with a higher prevalence and severity of hepatic fibrosis in observational studies.^[6,7]^ However, the evidence base relies exclusively on associational methods, such as logistic regression, Cox models, and propensity score matching, which cannot distinguish causal effects from residual confounding. MASLD and MetS are also linked bidirectionally in the natural history of the disease,^[8]^ which compounds the identification problem.

The nondiabetic MASLD subpopulation merits specific investigation because type 2 diabetes is an independent and potent driver of hepatic fibrogenesis.^[9]^ The exclusion of diabetic patients isolates the incremental fibrogenic contribution of the MetS composite and addresses the potential definitional circularity between MASLD (requiring ≥1 cardiometabolic risk factor) and MetS (requiring ≥3 of 5 components).^[1]^

Double machine learning (DML) is a semiparametric causal inference framework that combines flexible ML estimation of nuisance functions with Neyman-orthogonal moment conditions and cross-fitting, producing root-n consistent estimates under weaker assumptions than traditional parametric methods.^[10]^ DML has seen limited application in hepatology research.

This study aimed to quantify the covariate-adjusted difference in noninvasive fibrosis-marker burden between the supra-threshold (≥3/5) and subthreshold (0–2/5) MetS phenotypes in nondiabetic adults with MASLD, using DML under an explicitly stated structural causal model. Because the 2 available noninvasive outcomes differ in their dependence on age, 3 co-primary outcomes were prespecified (fibrosis-4 index [FIB-4], age-residualized FIB-4 and vibration-controlled transient elastography) so that the sensitivity of the estimate to outcome definition could be assessed directly rather than inferred.

## 2. Methods

### 2.1. Study design and data source

This cross-sectional causal inference study used data from the National Health and Nutrition Examination Survey (NHANES) 2017 to 2020 pre-pandemic cycle,^[11]^ with an external consistency assessment using the Tam Anh General Hospital cohort (Ho Chi Minh City, Vietnam). The study was designed using the structural causal model.^[12]^

The target parameter is a covariate-standardized stratum contrast rather than an interventional effect: Δ = *E*_*X*[*E*(*Y* | *A* = 1, *X*) − *E*(*Y* | *A* = 0, *X*)], where *A* indicates the suprathreshold MetS phenotype (≥3/5 Harmonized components), *Y* indicates fibrosis-marker positivity, and *X* is the confounder set defined below, with the expectation taken over the covariate distribution of the analytic sample. We deliberately do not write this as *P*(*Y* = 1 | do(*A* = 1)) −*P*(*Y* = 1 | do(*A* = 0)). As set out under “Estimand definition and its structural limits,” MetS is a composite of the same physiological axis that defines MASLD and cannot be set independently of its own constituents, so a do-operator formulation would assert an intervention this design does not support.

The study was preregistered on the Open Science Framework (DOI: 10.17605/OSF.IO/6XKMF) prior to manuscript finalization (post hoc relative to data analysis). This report follows the Strengthening the Reporting of Observational Studies in Epidemiology guidelines for cross-sectional studies.^[13]^

### 2.2. Participants

NHANES is a nationally representative cross-sectional survey conducted by the National Center for Health Statistics.^[11]^ The 2017 to 2020 pre-pandemic cycle was selected as the most recent cycle incorporating vibration-controlled transient elastography (VCTE).^[14]^ Eligible participants were adults aged 18 to 80 years who completed the mobile examination center visit with valid VCTE measurements. A VCTE examination was considered valid when the examination status variable indicated a complete examination, a median stiffness and a median controlled attenuation parameter were both recorded, and the interquartile range to median ratio for stiffness was ≤30%. The inclusion criteria were: hepatic steatosis defined by controlled attenuation parameter ≥248 dB/m, corresponding to steatosis grade ≥*S*1;^[15]^ fasting blood samples (≥8 hours); and complete data for all 5 MetS components.

The exclusion criteria were: diabetes mellitus, defined as meeting any 1 of fasting plasma glucose (FPG) ≥7.0 mmol/L, hemoglobin A1c ≥6.5%, or current antidiabetic medication use; excessive alcohol consumption (>30 g/d for men, >20 g/d for women), consistent with MASLD consensus;^[1]^ hepatitis B surface antigen or hepatitis C antibody positivity; and incomplete confounder data.

Medication use was classified from the prescription-medication file by matching generic drug names against prespecified class lists for statins, antidiabetic agents, and antihypertensive agents. Alcohol intake was derived by multiplying the reported drinking-frequency category by the reported usual number of drinks per drinking day, converted to grams per day at 14 g of ethanol per standard drink.

Of 1402 eligible participants, 96 (6.8%) were excluded for missing confounder data. Complete-case analysis was used given <10% missingness.

Among the final 1306 participants, 45 (3.4%) had no active MetS component. We reexamined their MASLD eligibility against the 2023 cardiometabolic criteria.^[1]^ Twenty-five met the adiposity criterion (body mass index ≥25 or ≥23 kg/m^2^ in participants of Asian ancestry or waist circumference ≥94 cm in men and ≥80 cm in women) and 1 further participant met the dysglycemia criterion, giving 26 who qualified. Nineteen participants (1.5% of the analytic sample) met no cardiometabolic criterion and would be classified as cryptogenic steatotic liver disease rather than MASLD under the 2023 nomenclature.^[1]^ All nineteen fall in the MetS− group. We retained them in the primary analysis to preserve the prespecified sample definition and report a sensitivity analysis excluding them.

The Tam Anh Hospital cohort comprised 106 consecutive nondiabetic MASLD patients identified by retrospective review of clinical records. The records covered attendances between January 2023 and December 2024 and included acoustic radiation force impulse elastography. This cohort was used solely for descriptive comparison of effect direction with the NHANES estimate. The record review itself was conducted between June 2023 and December 2024, within the research period covered by the ethics approval; no data were extracted before approval was granted.

### 2.3. Exposure definition

Metabolic syndrome was defined per the 2009 harmonized criteria,^[5]^ requiring ≥3 of 5 components: central obesity by ethnicity-specific waist circumference;^[16]^ triglycerides ≥1.7 mmol/L or lipid-lowering treatment; high-density lipoprotein (HDL) cholesterol <1.0 mmol/L (men) or <1.3 mmol/L (women); blood pressure ≥130/85 mm Hg or antihypertensive treatment; FPG ≥5.6 mmol/L (but <7.0 mmol/L). Component counts (0–5) were recorded for dose–response analysis.

### 2.4. Estimand definition and its structural limits

We state 3 constraints on the exposure before reporting the estimate.

#### 2.4.1. The exposure is not externally manipulable

Metabolic syndrome is a threshold applied to a composite of 5 correlated physiological measurements, all 5 of which (waist circumference, triglycerides, HDL cholesterol, blood pressure, and fasting glucose ≥5.6 mmol/L) are also qualifying cardiometabolic criteria for MASLD itself. Exposure and disease state therefore lie on a shared biological axis. Excluding diabetes and contrasting ≥3 against 0 to 2 components reduces definitional overlap but does not convert the exposure into an external intervention. The estimand is a contrast between 2 phenotypic strata of 1 severity continuum and should be read as such: it describes how much more fibrosis-marker burden covariate-comparable individuals in the suprathreshold stratum carry, not what would happen if MetS were removed.

#### 2.4.2. Multiple versions of treatment

Dichotomizing a 5-level ordinal count at ≥3 makes the exposure compound: *A* = 1 is realized by any of *C*(5, 3) + *C*(5, 4) + *C*(5, 5) = 16 distinct component configurations, and *A* = 0 by a further 16. The consistency assumption in its strict form is therefore violated, and the estimate is a weighted average over the configuration mix present in this sample. All 32 configurations were observed (Table S1, Supplemental Digital Content 1), so the estimate averages over the entire configuration space rather than a truncated part of it, but it will not transport unchanged to a population with a different mix. The Shapley decomposition reported below is the direct response to this limitation: it attributes the compound effect to individual components rather than leaving the composite unresolved.

#### 2.4.3. Selection into MASLD is a collider

Because MASLD requires at least 1 cardiometabolic abnormality and all 5 MetS components qualify, restricting the analysis to MASLD conditions on a common effect of the exposure and of unmeasured determinants of hepatic steatosis. We quantified this dependence directly. Within the fasting subsample from which the analytic sample was drawn, the probability of entering the analytic sample rose 3.5-fold with component count, from 12.9% at 0 components to 45.5% at 3, and participants meeting the exposure definition were substantially more likely to enter the study than those who did not (40.7% vs 30.7%; risk ratio 1.33; risk difference (RD) 10.0 percentage points; *P* < .001). Central obesity was the dominant channel (odds ratio 3.46 for selection), with triglycerides 2nd (1.47) and the remaining components effectively null; adding the components to a selection model containing the confounder set raised its discrimination from an area under the curve (AUC) of 0.602 to 0.696 (Fig. S2, Supplemental Digital Content 2). Forty-five participants (3.4%) entered with 0 active MetS components; 26 of them qualified for MASLD on a non-MetS cardiometabolic criterion (25 by adiposity, 1 by dysglycemia) and 19 met none, as reported under Section 2.2, so the contrast retains a genuinely unexposed comparison stratum. We address the measured component of this problem by inverse-probability-of-selection weighting (below); selection operating through unmeasured steatosis determinants cannot be corrected by weighting.

### 2.5. Outcome definitions

Three co-primary outcomes were prespecified. The 1st was elevated FIB-4–defined fibrosis risk (FIB-4 ≥1.3), where FIB-4 = (age × aspartate aminotransferase [AST])/(platelet × √alanine aminotransferase [ALT]).^[17]^ We describe this threshold as identifying elevated fibrosis risk rather than “significant fibrosis,” because FIB-4 <1.3 is validated as a rule-out threshold with high negative predictive value for advanced fibrosis, whereas values ≥1.3 denote an indeterminate-or-high risk category with limited positive predictive value in nondiabetic MASLD.^[18]^ The threshold is endorsed by the 2023 American Association for the Study of Liver Diseases Practice Guidance.^[19]^

The 2nd and 3rd co-primary outcomes were chosen to reduce or remove FIB-4’s age dependence (*r* = 0.468 between age and the FIB-4 score). The 1st of these, age-residualized FIB-4, is defined as the residuals from a linear regression of the score on age with the same prevalence threshold applied; it removes the linear component of that dependence but not the multiplicative structure through which age enters the formula. The 2nd, FibroScan ≥ 7.9 kPa, validated in biopsy-confirmed NAFLD (area under the receiver operating characteristic curve 0.80; ≥*F*2),^[20]^ contains no age term.

Because FIB-4 depends on both age and platelet count, whereas the residualized outcome does so only in part and elastography not at all, we prespecified the interpretive rule that governs the Sections 3 and 4: where the 3 estimates disagree, the most conservative is the 1 carried into clinical interpretation. The secondary outcome was severe fibrosis (FIB-4 ≥ 2.67).

Prediabetes was defined as FPG 5.6 to 6.9 mmol/L or hemoglobin A1c 5.7% to 6.4% without diabetes, per American Diabetes Association criteria,^[21]^ and was a prespecified effect modifier.

### 2.6. Confounder selection and propensity assessment

Confounders were selected from an expert-specified directed acyclic graph (DAG),^[12]^ validated against a data-driven completed partially DAG (CPDAG) via the Peter–Clark algorithm.^[22,23]^ The adjustment set comprised age (continuous), sex (binary), race/ethnicity (six NHANES categories entered as 5 indicator variables with Mexican American as reference), current smoking (binary), alcohol intake (drinks/wk, continuous), and statin use (binary). Potential mediators (body mass index, waist circumference, blood pressure, glucose, triglycerides, HDL, AST, ALT, gamma-glutamyl transferase, albumin, and platelet count) were excluded to preserve the total estimated effect.

Constraint-based discovery was run with temporal background knowledge forbidding any edge into age or sex, since neither can be caused by a downstream variable. Without that constraint, the algorithm returns temporally impossible orientations, and we report the comparison in the Section 3 rather than the unconstrained output alone.

### 2.7. Causal identification assumptions

Interpretation of DML estimates as effect estimates requires 3 assumptions:^[10,12]^

#### 2.7.1. Conditional ignorability

*Y*(*a*) ⊥ *A* | *X*, supported by DAG-guided confounder selection and negative control analyses. Residual confounding was assessed via *E* value, Rosenbaum Γ, and Oster δ.

#### 2.7.2. Positivity

0 < *P*(*A* = 1 | *X* = *x*) < 1 for all *x*. This was assessed using propensity score distributions and cross-validated AUC, and addressed by trimming. Statins (standardized mean difference [SMD] = 0.901) posed a near-structural violation, addressed by a dedicated sensitivity analysis.

#### 2.7.3. Stable unit treatment value

Each individual’s outcome depends only on their own MetS status, without interference; plausible for an individual-level metabolic phenotype. The no-multiple-versions component is, however, violated: as set out above, the ≥3/5 definition admits 16 distinct component configurations, so the estimate averages over versions rather than isolating 1. We report the configuration mix and decompose the composite by Shapley attribution rather than assuming the violation away.

#### 2.7.4. What DML does and does not deliver

DML addresses confounding: it estimates nuisance functions flexibly and, through Neyman orthogonality and cross-fitting, yields valid inference for the adjusted contrast. It contributes nothing to establishing temporal ordering. No orthogonalization, cross-fitting, or doubly robust construction can recover a time order that cross-sectional data do not contain, and the possibility that hepatic disease precedes and produces metabolic disturbance is not addressed by any analysis in this manuscript. Temporal ordering must come from the design; no estimator can supply it.

### 2.8. Causal estimation methods

The primary estimator was DML, combining flexible ML estimation of nuisance functions with Neyman-orthogonal moment conditions and *K*-fold cross-fitting.^[10]^ We used LinearDML (EconML^[24]^) with light gradient boosting machine (LightGBM)^[25]^ base learners: the LightGBM regressor class (LGBMRegressor) for the outcome model and the LightGBM classifier class (LGBMClassifier) for the treatment model, with discrete_treatment = True. Five-fold cross-fitting was applied with a fixed random seed (42).

The final-stage parameterization is reported explicitly because it materially affects the estimate. In the primary specification, the confounders enter as effect modifiers, so the final stage fits a linear conditional average treatment effect surface which is then averaged over the sample. An alternative constant-effect parameterization, in which the confounders enter only as controls, yields the canonical partially linear estimand. We retained the linear-conditional average treatment effect (CATE) parameterization as primary because the best linear predictor calibration test indicated significant effect heterogeneity (*a*_2_ = 3.42; *P* < .001), which a constant-effect specification cannot represent. The cost is precision: the constant-effect estimator has approximately half the standard error (SE) and yields a point estimate about 40% lower. Both are reported (Table 1).

**Table 1:** Specification stability of the primary contrast (N = 1306).

Perturbation	Adjusted risk difference	95% CI
Primary: linear-CATE final stage, 1-hot race, LightGBM, 5-fold, seed 42	**0.153**	0.057–0.250
Final stage: constant effect	0.095	0.050–0.140
Race coding: numeric	0.122	0.027–0.217
Cross-fitting: 10-fold	0.158	0.043–0.273
Random seed: 10 seeds, mean (range)	0.158	0.083–0.210
Nuisance learners: random forest	0.087	−0.035 to 0.209
Nuisance learners: regularized linear/logistic	0.163	0.094–0.233
Adjustment set: statin excluded	0.078	0.031–0.126
Weighting: inverse-probability-of-selection	0.126	0.003–0.250
Weighting: survey, P95-trimmed (Fay BRR)	0.142	0.023–0.261

Range across specifications: 0.078 to 0.163. The random-forest interval includes zero.

Bold indicates the primary specification.

BRR = balanced repeated replication, CATE = conditional average treatment effect, CI = confidence interval, LightGBM = light gradient boosting machine.

Hyperparameters were tuned via Optuna^[26]^ Bayesian optimization (30 trials per model). Nuisance model quality was evaluated via cross-validated AUC (propensity) and mean squared error (outcome).

The confirmatory estimator was augmented inverse probability weighting (AIPW),^[27,28]^ implemented as the linear doubly robust learner (LinearDRLearner) in EconML.^[24]^ The AIPW instability under indicator-encoded race (average treatment effect [ATE] = −0.113; SE = 0.354) reflects propensity model misspecification from sparse race-by-treatment cells; DML remained stable under both encoding schemes.

### 2.9. Interpretability of the causal machine-learning pipeline

We report interpretability along the 3 dimensions proposed by Mediouni et al: assessment of individual input features, traceability, and assessment of interactions between features.^[29]^

#### 2.9.1. Individual input features

Component-level attribution uses a cooperative-game decomposition in which the characteristic function is the DML effect estimate itself, not a predictive importance measure. For each of the 32 coalitions *S* ⊆ {waist, triglycerides, HDL, blood pressure, and glucose} we define the coalition treatment *A*_*S* = 1{all components in *S* present} and estimate *v*(*S*) = ATE_DML(*A*_*S*) by a full cross-fitted DML fit. The Shapley value of component *i* is φ_*i*_ = Σ_{*S* ⊆ N∖{*i*}} [|*S*|!(*k* − |*S*| − 1)!/k!]·[*v*(*S* ∪ {*i*}) −*v*(*S*)], with *k* = 5. These are effect-scale attributions measured in risk-difference units. Because φ is a fixed linear functional of the 32 coalition estimates and each estimate has a per-observation orthogonal score, the covariance of φ follows by the delta method from the empirical covariance of the stacked scores, giving analytic SEs without bootstrapping. By the efficiency property, Σφ equals *v*(N) (the contrast of all 5 components present vs not all present) which is a different estimand from the ≥3 vs 0 to 2 contrast; no rescaling between the 2 is defined, and none is applied. Shapley values decompose a compound exposure; they do not identify mediation, because they do not intervene on 1 component while holding the remainder at fixed levels.

#### 2.9.2. Traceability

The estimator is a 2-stage semiparametric procedure whose output is a sample mean. Within each of 5 cross-fitting folds, a LightGBM propensity model trained on the remaining folds produces *ê*(*X*) for held-out observations, and LightGBM outcome models trained separately on treated and untreated observations of the remaining folds produce μ̂_1_(*X*) and μ̂_0_(*X*). These enter the Neyman-orthogonal score ψ_*i*_ = μ̂_1_(*X*_*i*_) − μ̂_0_(*X*_*i*_) + *A*_*i*_{*Y*_*i*_ − μ̂_1_(*X*_*i*_)}/*ê*(*X*_*i*_) − (1 − *A*_*i*_){*Y*_*i*_ − μ̂_0_(*X*_*i*_)}/{1 − *ê*(*X*_*i*_)}; the estimate is the sample mean of ψ and its SE the sample standard deviation of ψ divided by √n. The gradient-boosted learners never act on the treatment effect directly. They supply 2 nuisance functions, and orthogonality makes the estimate 1st-order insensitive to error in either. Specification stability is reported in Table 1 across final-stage parameterization, race coding, effect estimator, propensity trimming, statin adjustment, survey weighting, number of cross-fitting folds, random seed, and nuisance learner family. All analysis code is deposited at https://github.com/drhientran/mets-masld-dml.

#### 2.9.3. Interactions between features

Beyond conditional average treatment effects, we estimate additive-scale interaction between component pairs by a factorial contrast. For components (*C*_1_, *C*_2_) we estimate 3 DML contrasts against the doubly-unexposed cell: (1, 0), (0, 1) and (1, 1), each versus (0, 0). We then form INT = ATE₍_1_,_1_₎ − ATE₍_1_,_0_₎ − ATE₍_0_,_1_₎, the risk-difference analogue of the relative excess risk due to interaction, with inference from the same stacked-score covariance. Fasting glucose × central obesity is the prespecified primary pair; the remaining 9 pairs are exploratory with Holm adjustment, and cells containing <25 participants are reported as non-estimable.

### 2.10. Treatment effect heterogeneity

CATE was estimated via CausalForestDML (honest estimation, 1000 trees, and minimum leaf 20).^[30,31]^ Heterogeneity was tested by best linear predictor calibration with heteroskedasticity-consistent (type 1) robust SE on cross-validated residuals. Seven subgroups were defined a priori in Protocol v2.0: age (<40, 40–59, ≥60), sex, and prediabetes status. Bootstrap 95% percentile intervals over 200 resamples, with *P* values from a normal approximation to the bootstrap SE. CATE was interpreted directionally. Robustness was assessed using T-, S-, X-Learner^[32]^ and R-Learner (NonParamDML),^[10]^ all with the LightGBM regressor (LGBMRegressor).

### 2.11. Sensitivity analyses

Analyses in this subsection through “Statin sensitivity” were prespecified in Protocol v2.0. The analyses that follow, from “Age-stratified analysis” onward, were performed at the request of peer reviewers and are post hoc.

#### 2.11.1. Negative controls

Outcome: adult height; exposure: simulated birth quarter.^[33,34]^

#### 2.11.2. Unmeasured confounding

*E* value;^[35]^ Rosenbaum Γ-bounds;^[36]^ Oster δ.^[37]^

#### 2.11.3. Shapley decomposition

32 coalition DMLs, as specified above.^[38]^

#### 2.11.4. Propensity trimming

Three thresholds: (0.01, 0.99), (0.05, 0.95), (0.10, 0.90).

#### 2.11.5. Statin sensitivity

The primary analysis was repeated without statins (SMD = 0.901).

#### 2.11.6. Age-stratified analysis

Because FIB-4 incorporates age multiplicatively while FibroScan does not, we estimated stratum-specific contrasts within the age bands used for subgroup analysis (<40, 40–59, ≥60 years) for all 3 co-primary outcomes, with a test of effect modification comparing the oldest and youngest strata and a 2-degree-of-freedom Cochran test of homogeneity. We fixed the interpretation in advance. If the contrast is present in the youngest stratum, where the age term has the least room to operate, a pure formula artifact is unlikely. If it is absent below 40 and rises monotonically with age, the primary finding must be reread as substantially age-driven. Strata with <10 outcome events are reported as non-estimable. In weighing the stratified estimates, the comparison that bears on the age question is between the 2 FIB-4 outcomes and the elastography outcome, which contains no age term, because linear residualization leaves the multiplicative age structure of FIB-4 in place; the stratified estimates for the age-residualized outcome are therefore treated as supporting evidence only.

#### 2.11.7. Platelet-channel analysis

Because FIB-4 places platelet count in the denominator, reverse causation through splenomegaly-associated thrombocytopenia would inflate the estimate. We report platelet distribution by exposure status, reestimate excluding participants with platelets below 150 × 10^9^/L, and reestimate with FIB-4 recomputed holding platelet count at the cohort median.

#### 2.11.8. Selection weighting

A logistic selection model for entry into the analytic sample was fitted across the full fasting subsample reconstructed from the raw public-use files, and the primary contrast was reestimated with stabilized inverse-probability-of-selection weights.

#### 2.11.9. Survey-weighted replication

The survey-weighted point estimate was obtained from a full-sample weighted DML fit using NHANES fasting subsample weights, raw and trimmed at the 95th percentile. Variance was estimated by Fay-adjusted balanced repeated replication (*k* = 0.3; 32 Hadamard replicates over 24 strata with 2 primary sampling units each) and, as a conservative comparison, by a Rao–Wu half-sample bootstrap with 200 replicates. Replicates supply the variance only; the point estimate is not the replicate mean.

#### 2.11.10. Minimum detectable effect

For each co-primary outcome, and separately within each age stratum, we report the smallest effect detectable at 80% power and 2-sided α = 0.05, under both parameterizations, so that null results can be read against what the analysis was able to detect.

#### 2.11.11. MASLD eligibility

We re-derived the 2023 cardiometabolic criteria for every participant and repeated the primary analysis excluding those who met none of them.

### 2.12. External consistency

Tam Anh (N = 106): the crude RD was compared with the NHANES estimates for directional concordance. Independent causal estimation was not performed because of insufficient sample size.

### 2.13. Pipeline validation

The pipeline was validated on synthetic data (N = 5000) with a known data-generating process. The true marginal ATE was computed via Monte Carlo integration with 100,000 draws. Four criteria were required: DML confidence interval (CI) covers true ATE; AIPW within 0.03; and both negative controls null.

### 2.14. Ethical considerations

This study was conducted in accordance with the Declaration of Helsinki. The NHANES 2017 to 2020 cycle was approved by the National Center for Health Statistics Research Ethics Review Board (protocol #2018-01), and all participants provided written informed consent.^[11]^ Ethics approval for the Tam Anh cohort was granted on June 2, 2023 by the Biomedical Research Ethics Committee of the University of Medicine and Pharmacy, Hue University (no. H2023/372), the academic institution supervising the investigator, under an expedited review procedure, for a research period running from June 2023 to December 2024. The study was a retrospective review of existing clinical records, and all data extraction took place within that research period. Individual informed consent was not required for this component. The study was a retrospective review of preexisting clinical records: no participant was contacted and no participant underwent any procedure for the purposes of this study. Records were de-identified at extraction, before any analysis was performed, and the analytic dataset carries a sequential study number in place of any hospital identifier and contains no dates, names or contact details, so that the analysis was conducted on data from which individuals cannot be identified. Institutional permission for access to clinical records was granted by Tam Anh General Hospital. Secondary NHANES analysis is exempt per 45 CFR §46.104(d)(4).

### 2.15. Sample size considerations

The sample of 1306 (760 MetS+, 546 MetS−) exceeded the minimum of ~500 for moderate-dimensional semiparametric causal estimation,^[10]^ providing >80% power to detect a RD ≥0.08 at α = 0.05, given a baseline fibrosis-marker prevalence of 13.2% and 5-fold cross-fitting.

### 2.16. Software, reproducibility, and statistical expertise

All analyses were performed in Python version 3.12 (Python Software Foundation) using EconML version 0.15 or later (Microsoft Research),^[24]^ LightGBM version 4.0 or later (Microsoft Corporation),^[25]^ Optuna version 3.5 or later (Preferred Networks Inc.),^[26]^ and causal-learn (Carnegie Mellon University).^[23]^ The random seed was 42 throughout. Hierarchical testing: Tier 1 (confirmatory, α = 0.05): primary DML FIB-4; Tier 2 (Holm α = 0.01): severe fibrosis + Shapley; Tier 3 (exploratory): CATE, meta-learners, FibroScan, and component interactions. The analysis code, covering data construction, all estimators, all robustness analyses and the scripts that generate every table and figure, is deposited at https://github.com/drhientran/mets-masld-dml and archived with a persistent identifier at https://doi.org/10.5281/zenodo.21775306 (version v2.0.1), so that any value in this manuscript can be traced to the code that produced it.

Statistical expertise was provided from within the author group, as recorded in the Acknowledgments; clinical interpretation was provided by the corresponding author.

## 3. Results

### 3.1. Participant selection

Of the 15,560 adults examined in the NHANES 2017 to 2020 pre-pandemic cycle, 1306 met all inclusion criteria (Fig. 1). Sequential exclusions were: absence of valid VCTE (n = 8262), non-fasting (n = 3650), controlled attenuation parameter < 248 dB/m (n = 1236), diabetes mellitus (n = 627), viral hepatitis B or C (n = 173), incomplete MetS components (n = 210), and incomplete confounders (n = 96). The final sample comprised 760 participants with MetS (58.2%) and 546 without (41.8%).

**Figure 1.**
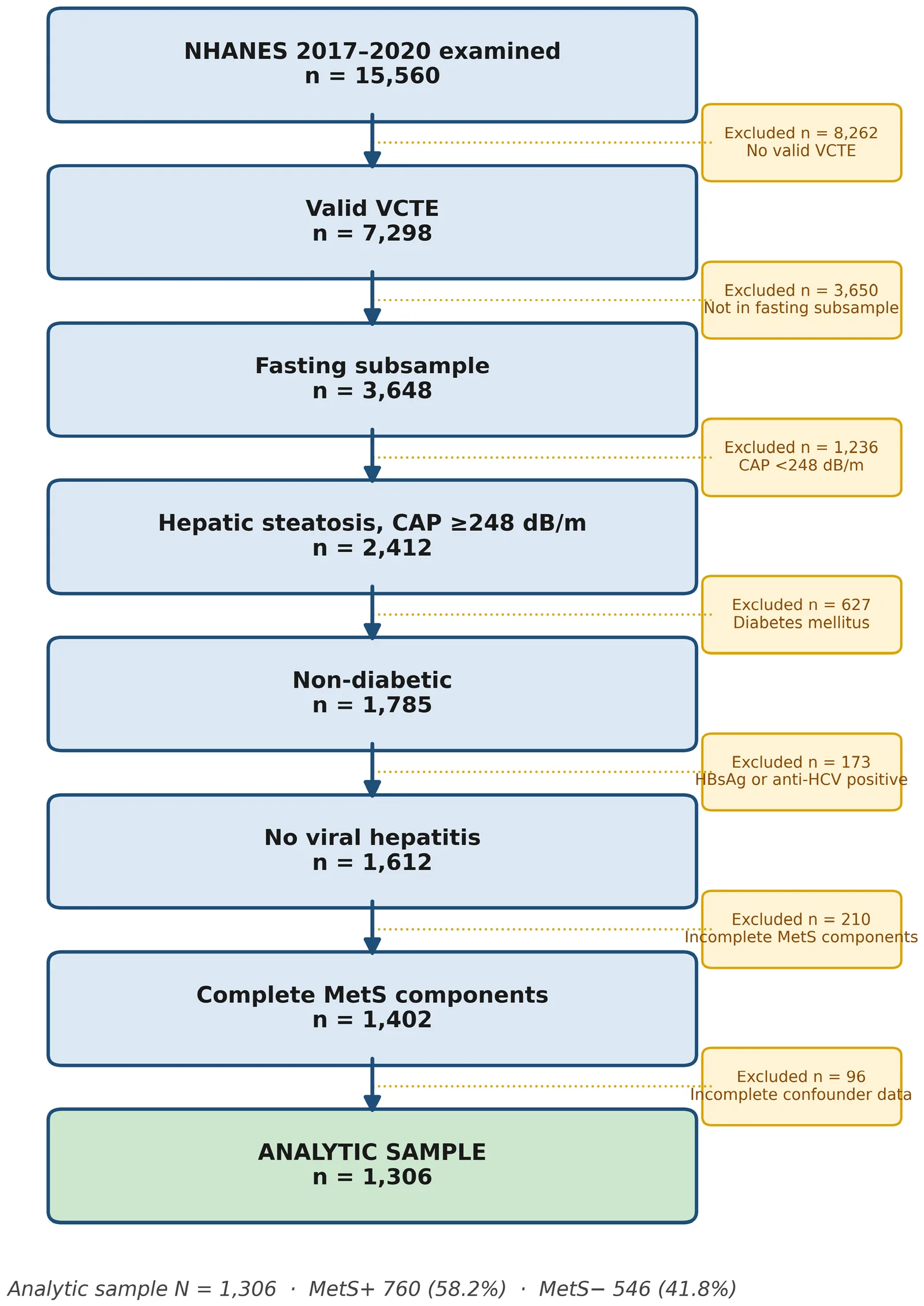
Participant flow. NHANES 2017 to 2020 pre-pandemic. Analytic sample N = 1306 of 15,560 examined participants; exclusions and reasons shown at each stage. MetS +760 (58.2%), MetS −546 (41.8%). CAP = controlled attenuation parameter, HBsAg = hepatitis B surface antigen, HCV = hepatitis C virus, MetS = metabolic syndrome, NHANES = National Health and Nutrition Examination Survey, VCTE = vibration-controlled transient elastography.

### 3.2. Baseline characteristics

Baseline characteristics by MetS status are in Table 2. The MetS+ group was older (53.9 ± 16.0 vs 42.5 ± 16.2 years; SMD = 0.715) and had a higher prediabetes prevalence (90.0% vs 50.4%; SMD = 0.960). Sex, race/ethnicity, smoking status, and alcohol intake were balanced (SMDs < 0.11). Statin use showed extreme imbalance: 223/760 (29.3%) versus 1/546 (0.2%) (SMD = 0.901), indicating near-complete positivity violation, addressed by dedicated sensitivity analysis. Platelet count was similar between groups (250.1 ± 66.3 vs 248.5 ± 57.5 × 10^9^/L; *P* = .66), with counts below 150 × 10^9^/L in 27 (3.6%) and 12 (2.2%), respectively.

**Table 2:** Baseline characteristics by metabolic syndrome status, National Health and Nutrition Examination Survey 2017 to March 2020 (N = 1306).

Variable	All (N = 1306)	MetS + (N = 760)	MetS − (N = 546)	SMD
Age, yr	49.1 ± 17.0	53.9 ± 16.0	42.5 ± 16.2	0.715
Male, n (%)	667 (51.1)	371 (48.8)	296 (54.2)	0.108
Race and ethnicity, n (%)				
Mexican American	191 (14.6)	97 (12.8)	94 (17.2)	0.125
Other Hispanic	129 (9.9)	85 (11.2)	44 (8.1)	0.106
Non-Hispanic White	514 (39.4)	305 (40.1)	209 (38.3)	0.038
Non-Hispanic Black	265 (20.3)	154 (20.3)	111 (20.3)	0.002
Non-Hispanic Asian	140 (10.7)	85 (11.2)	55 (10.1)	0.036
Other or multiracial	67 (5.1)	34 (4.5)	33 (6.0)	0.070
Current smoker, n (%)	210 (16.1)	127 (16.7)	83 (15.2)	0.041
Alcohol, drinks/wk, mean	2.1	2.1	2.2	0.052
Alcohol, drinks/wk, median [IQR]	0.2 [0.0–2.0]	0.2 [0.0–2.0]	0.3 [0.0–2.5]	–
Statin use, n (%)	224 (17.2)	223 (29.3)	1 (0.2)	0.901*
Platelet count, ×10^9^/L	249.4 ± 62.7	250.1 ± 66.3	248.5 ± 57.5	0.025
Platelets <150 × 10^9^/L, n (%)	39 (3.0)	27 (3.6)	12 (2.2)	0.081
FibroScan, kPa, median [IQR]	5.1 [4.2–6.2]	5.2 [4.3–6.4]	4.9 [4.2–5.9]	0.202
FIB-4 ≥ 1.3, n (%)	331 (25.3)	259 (34.1)	72 (13.2)	0.507
FibroScan ≥ 7.9 kPa, n (%)	135 (10.3)	95 (12.5)	40 (7.3)	0.174
Prediabetes, n (%)	959 (73.4)	684 (90.0)	275 (50.4)	0.960*

Continuous variables are mean ± SD unless marked as median [IQR]. MetS+ denotes ≥3 of 5 harmonized components; MetS− denotes 0 to 2. SMD, standardized mean difference; an asterisk marks SMD > 0.9, reflecting near-complete separation rather than imbalance amenable to adjustment. The alcohol row in the previous version reported the mean while labeling it a median; both are now given, since the distribution is heavily right-skewed and the mean of 2.1 drinks/week lies outside its own interquartile range. The race and ethnicity block previously used 4 categories, merging Mexican American, Other Hispanic, and Other or multiracial participants into a single row labeled “Hispanic (other)”; all 6 NHANES categories are now listed separately, which in particular stops 67 participants recorded as other or multiracial from being reported as Hispanic. Platelet count is added because it enters the FIB-4 denominator and is therefore relevant to the interpretation of the primary outcome (Table 1, platelet-neutralized specification).

FIB-4 = fibrosis-4 index, IQR = interquartile range, MetS = metabolic syndrome, SMD = standardized mean difference.

### 3.3. MASLD–MetS definitional overlap

Among the 1306 participants, 45 (3.4%) had 0 components, 501 (38.4%) had 1 to 2 (subthreshold), and 760 (58.2%) had ≥3 (Fig. 2). Fibrosis-marker prevalence showed a monotonic gradient: 6.7%, 13.8%, and 34.1% (*P* for trend <.001).

**Figure 2.**
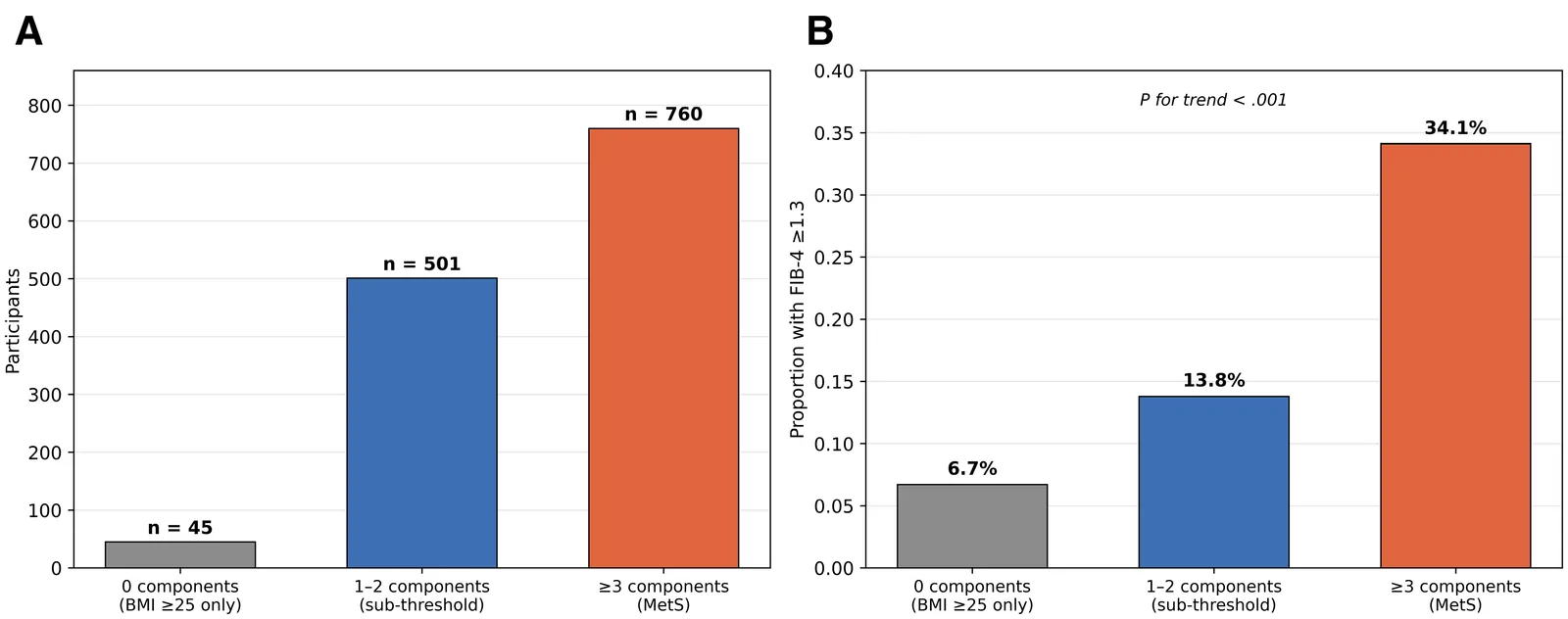
MASLD–MetS definitional overlap and fibrosis-marker prevalence by MetS component count. N = 1306 (0 components n = 45; 1–2 components n = 501; ≥3 components n = 760). (A) Group sizes. (B) Proportion with FIB-4 ≥ 1.3 (3/45, 69/501, 259/760); *P* for trend <.001. BMI = body mass index, FIB-4 = fibrosis-4 index, MASLD = metabolic dysfunction–associated steatotic liver disease, MetS = metabolic syndrome.

### 3.4. Estimand diagnostics

All 32 component configurations were observed (Table S1, Supplemental Digital Content 1), so the compound exposure is realized across its full configuration space. Selection into the analytic sample was strongly exposure-dependent: within the fasting subsample, the probability of entry rose from 12.9% at zero components to 45.5% at 3, and was 40.7% among participants meeting the exposure definition versus 30.7% among those not meeting it (risk ratio 1.33; RD 10.0 percentage points; *P* < .001; Fig. S2, Supplemental Digital Content 2). Central obesity carried an odds ratio of 3.46 for selection; adding the components to a selection model containing the confounder set raised discrimination from an AUC of 0.602 to 0.696.

### 3.5. DAG validation

The expert-specified DAG was compared with a data-driven CPDAG learned via the Peter–Clark algorithm (α = 0.05, Fisher’s *z*) on 10 variables (Fig. 3). Run without background knowledge, the algorithm returned temporally impossible orientations; statin use, MetS, AST, ALT, and FIB-4 each oriented as causes of age and oriented the MetS–FIB-4 edge in the reverse direction. That output does not support a directional claim, and we do not make 1 from it. Two constraints were imposed: nothing may cause age or sex, and FIB-4, being a deterministic arithmetic function of age, AST, ALT, and platelet count, may not cause any other variable. Together these removed every temporally impossible edge and oriented the MetS–FIB-4 edge in the hypothesized direction. The 2nd constraint matters. With only the temporal constraint imposed, the algorithm orients that edge in reverse; this reflects the behavior of the search and says nothing about the true direction. FIB-4 is a deterministic function of age, AST, ALT, and platelet count, so those variables stand in exact functional relation to the outcome and violate the faithfulness assumption on which constraint-based discovery depends; edges among them are artifacts of construction. Second, the background knowledge forbids only edges directed into age and sex; it places no constraint on the MetS–FIB-4 pair, which is oriented indirectly through the orientation–propagation rules that follow from those constraints. The orientation is therefore derived rather than assumed, but it is conditional on the temporal ordering we imposed. The CPDAG is therefore reported as a descriptive check on the adjacency structure of the expert DAG, not as independent causal evidence.

**Figure 3.**
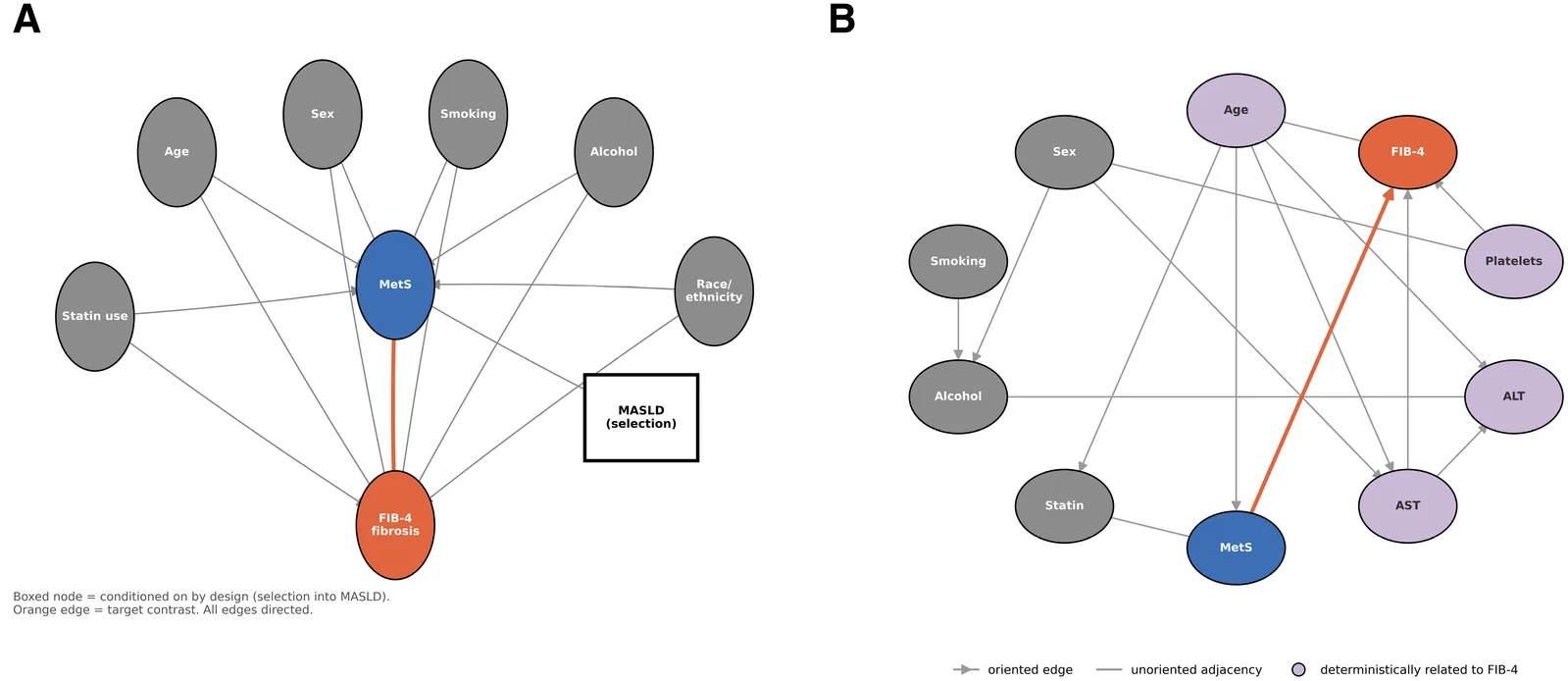
(A) Expert-specified directed acyclic graph, with directed edges, MASLD shown as a boxed node conditioned on by design, and unmeasured steatosis determinants shown as a dashed node. (B) Completed partially directed acyclic graph learned by the PC algorithm (α = 0.05, Fisher’s *z*) over 10 variables, constrained so that nothing may cause age or sex and so that FIB-4, a deterministic function of 4 of the other variables, may not cause any of them. Solid arrowheads denote oriented edges; plain lines denote unoriented adjacencies. Without the constraint, the algorithm returns temporally impossible orientations (statin, MetS, AST, ALT, and FIB-4 each as causes of age) and orients the MetS–FIB-4 edge in reverse. FIB-4 is a deterministic function of age, AST, ALT, and platelet count, so edges among those variables violate faithfulness and are not interpretable as discoveries. N = 1306. ALT = alanine aminotransferase, AST = aspartate aminotransferase, FIB-4 = fibrosis-4 index, MASLD = metabolic dysfunction–associated steatotic liver disease, MetS = metabolic syndrome, PC = Peter–Clark.

### 3.6. Primary contrast

Under the primary specification, the covariate-standardized contrast between the suprathreshold and subthreshold MetS phenotypes for FIB-4 ≥ 1.3 was 0.153 (95% CI, 0.057–0.250; SE = 0.049; *P* = .002; *E* value 3.75, lower confidence bound 2.22; Table 3). On the observed baseline prevalence of 13.2% in the subthreshold group, this corresponds to a prevalence ratio of approximately 2.2.

**Table 3:** Covariate-adjusted stratum contrasts (≥3 vs 0 to 2 metabolic syndrome components) for fibrosis markers.

Method	Adjusted risk difference	SE	95% CI	*P*	*E* value
DML, FIB-4 (primary)	0.153	0.049	0.057–0.250	.002	3.75/2.22
AIPW (numeric race)	0.132	0.020	0.093–0.172	<.001	–
AIPW (indicator race)	−0.113	0.354	−0.807 to 0.580	.75	–
DML, FibroScan	0.001	0.049	−0.094 to 0.097	.98	–
DML, FIB-4 severe (≥2.67)	0.019	0.018	−0.017 to 0.055	.30	–

The column previously headed “ATE” is now headed “Adjusted risk difference,” because the quantity estimated is a covariate-standardized contrast between 2 strata of a compound exposure rather than the effect of an intervention. Values are unchanged. *E* values are given for the point estimate and for the confidence-limit closest to the null.

AIPW = augmented inverse probability weighting, CI = confidence interval, DML = double machine learning, FIB-4 = fibrosis-4 index, SE = standard error.

The doubly robust AIPW estimator agreed under numeric race coding (0.132; 95% CI, 0.093–0.172) but was unstable under indicator encoding (−0.113; SE = 0.354), reflecting propensity model misspecification from sparse race-by-treatment cells rather than substantive disagreement. DML was stable under both encodings, and we report the indicator-encoded DML estimate as primary because it retains the full race stratification without inducing that instability.

### 3.7. Age-dependency and outcome-measure sensitivity

Using the original FIB-4 score, the contrast was 0.153 (*P* = .002). After regressing FIB-4 on age and applying the same prevalence threshold to the residuals, it decreased to 0.091 (95% CI, −0.018 to 0.201; *P* = .10). Using FibroScan ≥ 7.9 kPa, the estimate was 0.001 (95% CI, −0.094 to 0.097; *P* = .98). Results are summarized in Table 4 and Figure 4A.

**Table 4:** Age-dependency sensitivity analysis.

Outcome	Age dependence	Adjusted risk difference	95% CI	*P*
FIB-4 ≥ 1.3 (original)	Direct (enters the formula)	0.153	0.057–0.250	.002
FIB-4, age-residualized	Partially removed (linear component only)	0.091	−0.018 to 0.201	.10
FibroScan ≥ 7.9 kPa	None	0.001	−0.094 to 0.097	.98

Values unchanged from the previous version. The 3 outcomes are ordered by how strongly the measure depends on age; the estimate falls monotonically from the original score, through linear residualization, which reduces the age dependence, to elastography, which has none. Linear residualization removes the linear component of the age dependence but not the multiplicative structure through which age enters the formula.

CI = confidence interval, FIB-4 = fibrosis-4 index.

**Figure 4.**
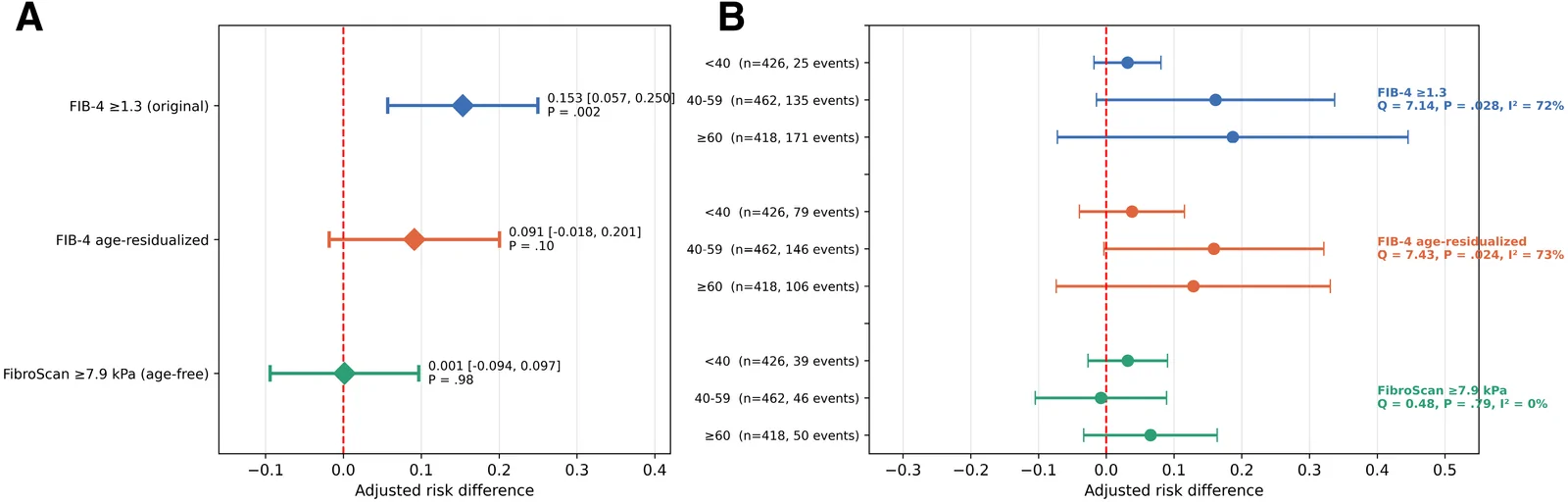
(A) Outcome-measure sensitivity: estimates for FIB-4, age-residualized FIB-4 and FibroScan. (B) Age-stratified estimates for all 3 outcomes with Cochran homogeneity statistics. Error bars: 95% CI. N = 1306; outcome events 331, 331, and 135 respectively. CI = confidence interval, FIB-4 = fibrosis-4 index.

The FibroScan interval excludes the FIB-4 point estimate of 0.153: the elastography data are not compatible with an effect of that magnitude. Second, this cannot be attributed to reduced power. Despite resting on 135 outcome events against 331 for FIB-4, the FibroScan analysis had essentially identical precision (SE 0.049 for both), and the minimum effect detectable at 80% power was 0.137 for FibroScan against 0.138 for FIB-4.

### 3.8. Age-stratified estimates

Stratum-specific contrasts for all 3 co-primary outcomes are given in Table 5 and Figure 4B. For FIB-4 the contrast was absent below age 40 (0.031; 95% CI, −0.018 to 0.081; *P* = .21; 25 events) and larger, though not statistically significant, at 40 to 59 (0.161; *P* = .072) and ≥ 60 (0.187; *P* = .16), with significant heterogeneity across strata (Cochran *Q* = 7.14, 2 df, *P* = .028, *I*^2^ = 72%). The oldest-versus-youngest difference was 0.092 (95% CI, −0.033 to 0.218; *P* = .15).

**Table 5:** Age-stratified contrasts for the 3 co-primary outcomes.

Outcome	Age	N	Events	Adjusted RD	95% CI	*P*
FIB-4 ≥ 1.3	<40	426	25	0.031	−0.018 to 0.081	.21
	40–59	462	135	0.161	−0.014 to 0.337	.072
	≥60	418	171	0.187	−0.072 to 0.446	.16
FIB-4 age-residualized	<40	426	79	0.038	−0.040 to 0.115	.34
	40–59	462	146	0.159	−0.003 to 0.321	.055
	≥60	418	106	0.129	−0.074 to 0.331	.21
FibroScan ≥ 7.9 kPa	<40	426	39	0.032	−0.027 to 0.090	.29
	40–59	462	46	−0.008	−0.105 to 0.089	.88
	≥60	418	50	0.065	−0.033 to 0.164	.19

Stratum estimates and intervals are from the primary linear-CATE specification. The Cochran homogeneity statistics and the effect-modification contrasts below are computed from the constant-effect stratum estimates, which are better behaved in strata of this size. Recomputed from the linear-CATE estimates tabulated above, they give *Q* = 3.13, *P* = .21, *I*^2^ = 36% for FIB-4, *Q* = 2.13, *P* = .35, *I*^2^ = 6% for the age-residualized outcome; and *Q* = 1.08, *P* = .58, *I*^2^ = 0% for FibroScan, so the absence of heterogeneity for the elastography outcome holds under both parameterizations. Constant-effect stratum values and stratum-specific minimum detectable effects are given in Table S2, Supplemental Digital Content 3. Minimum detectable effects at 80% power under the primary specification were 0.071 (<40), 0.251 (40–59), and 0.370 (≥60): the youngest stratum is the best powered, and the 2 older strata cannot resolve effects smaller than about a quarter. Effect modification (≥60 vs <40): FIB-4 0.092 (−0.033 to 0.218), *P* = .15; age-residualized 0.105 (−0.019 to 0.228), *P* = .096; FibroScan 0.024 (−0.070 to 0.118), *P* = .61. Cochran homogeneity across strata: FIB-4 *Q* = 7.14, *P* = .028, *I*^2^ = 72%; age-residualized *Q* = 7.43, *P* = .024, *I*^2^ = 73%; FibroScan *Q* = 0.48, *P* = .79, *I*^2^ = 0%. Under the constant-effect specification, the 40 to 59 and ≥60 strata reach significance for both FIB-4 outcomes (Table S2, Supplemental Digital Content 3).

CATE = conditional average treatment effect, CI = confidence interval, FIB-4 = fibrosis-4 index, RD = risk difference.

Taken alone, this pattern is consistent with an age artifact, and under the rule fixed in advance, the FIB-4 contrast must be read as substantially age-driven. The comparison that bears on the question is with the outcome that contains no age term. The age-independent elastography outcome showed no heterogeneity across strata under either parameterization: *Q* = 0.48, *P* = .79, *I*^2^ = 0% from the constant-effect stratum estimates, from which the Cochran statistics in Table 5 are computed, and *Q* = 1.08, *P* = .58, *I*^2^ = 0% from the linear-CATE estimates of 0.032, −0.008 and 0.065. The gradient is therefore specific to FIB-4 and is most economically explained by its age term. The age-residualized outcome reproduces the FIB-4 gradient (absent below 40 at 0.038, *P* = .34; 0.159 at 40–59; 0.129 at ≥ 60) with equivalent heterogeneity (*Q* = 7.43, *P* = .024, *I*^2^ = 73%), and under the constant-effect specification reaches significance in both older strata (0.179, *P* < .001; and 0.140, *P* = .007; Table S2, Supplemental Digital Content 3). Because linear residualization removes only the linear component of the age dependence, whereas age enters FIB-4 multiplicatively, the residualized score retains age structure, and this persistence is what the formula itself predicts; it cannot discriminate arithmetic from biology, and we treat it as supporting evidence that the 2 FIB-4 outcomes behave alike rather than as evidence against an age artifact. Precision does not follow event count here. Although the youngest stratum contains only 25 FIB-4 events, it is the most precisely estimated of the 3 (SE 0.025) and could detect an effect of 0.071 or larger at 80% power; its CI, which reaches only 0.081, therefore excludes the overall estimate of 0.153. The result below age 40 is therefore a null with adequate power behind it. The 2 older strata are the imprecise ones: under the primary parameterization, the smallest effects they could detect were 0.251 and 0.370, so their *P* values of .072 and .16 carry little information. Under the constant-effect parameterization, which is better behaved in strata of this size, all 3 are adequately powered (minimum detectable effects 0.070, 0.108, and 0.165; Table S2, Supplemental Digital Content 3) and the 2 older strata reach significance.

### 3.9. Treatment effect heterogeneity

The best linear predictor calibration test, estimated on cross-fitted residuals with heteroskedasticity-consistent (type 1) robust SEs, confirmed significant heterogeneity (*a*_2_ = 3.42; SE 0.47; *P* < .001; Table 6). The coefficient exceeded the ideal value of 1.0, indicating the causal forest amplified heterogeneity predictions relative to true variation, a recognized limitation with binary outcomes at moderate sample sizes. Subgroup CATE estimates are therefore interpreted directionally. All 7 subgroups showed positive effects with bootstrap intervals excluding 0, though the oldest stratum was the least precisely estimated (0.141; 95% CI, 0.037 to 0.287; *P* = .028). Effects were larger in the 2 older age groups and in men; prediabetes status did not meaningfully modify the effect.

**Table 6:** Conditional average treatment effect by subgroup, causal forest with 200 bootstrap resamples.

Subgroup	N	CATE	95% CI	*P*
Age <40	426	0.082	0.027–0.136	.003
Age 40–59	462	0.143	0.080–0.245	.001
Age ≥60	418	0.141	0.037–0.287	.028
Male	667	0.138	0.078–0.240	.001
Female	639	0.106	0.055–0.178	.001
Prediabetes	959	0.129	0.073–0.215	.001
No prediabetes	347	0.105	0.064–0.166	<.001

Intervals are bootstrap percentiles over 200 resamples; *P* values are from a normal approximation using the bootstrap standard error, because the percentile method cannot resolve below 2/*B* = 0.01 at this number of resamples. Race/ethnicity is 1-hot encoded, as in the primary analysis. BLP calibration on cross-fitted residuals with HC1 standard errors: *a*_2_ = 3.42 (SE 0.47; *P* < .001). The coefficient exceeds the ideal calibration value of 1.0, so subgroup magnitudes should not be used for clinical decision-making. Subgroup CATE values are averages of the causal-forest surface within each subgroup and are not the same quantity as the stratum-specific double machine learning estimates in Table 5, which refit the estimator within each stratum.

BLP = best linear predictor, CATE = conditional average treatment effect, CI = confidence interval, HC1 = heteroskedasticity-consistent (type 1), SE = standard error.

### 3.10. Meta-learner robustness

Four additional estimators confirmed the positive FIB-4 contrast (Table 7): T-Learner 0.136, S-Learner 0.127, X-Learner 0.132, R-Learner 0.115; 0.115 to 0.153 across all specifications, all positive. Including both DML specifications and AIPW under the original coding, 7 of the 8 estimator-specification combinations yielded consistent positive results.

**Table 7:** Meta-learner robustness comparison.

Method	Adjusted risk difference	% of DML
DML (primary)	0.153	Reference
DML (numeric race)	0.132	86%
AIPW (numeric race)	0.132	86%
T-learner	0.136	89%
S-learner	0.127	83%
X-learner	0.132	86%
R-learner	0.115	75%

All 6 methods share LightGBM nuisance models and the same adjustment set. Their agreement demonstrates robustness to the choice of effect estimator, not to nuisance specification; the latter is addressed in Table 1.

AIPW = augmented inverse probability weighting, DML = double machine learning LightGBM = light gradient boosting machine.

### 3.11. Component-level attribution

Shapley values with SEs, 95% CIs, and Holm-adjusted *P* values for all 5 components are in Table 8 and Figure 5. All 32 coalitions were estimable, with Shapley weight coverage of 1.00 for every component. Under the constant-effect specification, fasting glucose ranked highest (φ = 0.018; 95% CI, −0.013 to 0.049) followed by central obesity (0.006; −0.025 to 0.037); elevated blood pressure had the most negative value (−0.035; −0.073 to 0.002). No component’s contribution differed significantly from zero, and the smallest Holm-adjusted *P* value was .33. Under the linear-CATE specification, the same ordering emerged with larger magnitudes (fasting glucose 0.054, central obesity 0.042). The component ranking should therefore be read as descriptive rather than as evidence that particular components carry the effect. Σφ equals *v*(N) (the contrast of all 5 components present vs not all present), which is a different estimand from the ≥ 3 versus 0 to 2 contrast; the 2 are not related by any defined rescaling.

**Table 8:** Shapley decomposition of the estimated effect across metabolic syndrome components (N = 1306).

Component	φ	SE	95% CI	*P*	*P* (Holm)	Weight covered
Elevated fasting plasma glucose	0.018	0.016	−0.013 to 0.049	.26	>.99	1.00
Central obesity (waist)	0.006	0.016	−0.025 to 0.037	.69	>.99	1.00
Low HDL cholesterol	−0.002	0.016	−0.032 to 0.029	.92	>.99	1.00
Elevated triglycerides	−0.006	0.019	−0.042 to 0.031	.77	>.99	1.00
Elevated blood pressure	−0.035	0.019	−0.073 to 0.002	.066	.33	1.00
**Σφ = *v*(N**)	**−0.018**					

The constant-effect specification is the primary decomposition because it is the only parameterization for which valid joint inference across coalitions is available; the linear-CATE values below are a sensitivity analysis. 32 coalition DML fits, all estimable. Σφ equals *v*(N), the all-five-present contrast, which is a different estimand from the ≥3 vs 0 to 2 contrast. Under the linear-CATE specification, the ordering is unchanged with larger magnitudes (fasting glucose 0.054, central obesity 0.042, blood pressure 0.016, low HDL −0.018, triglycerides −0.035; Σφ = 0.059).

CATE = conditional average treatment effect., CI = confidence interval, DML = double machine learning, HDL = high-density lipoprotein, SE = standard error.

**Figure 5.**
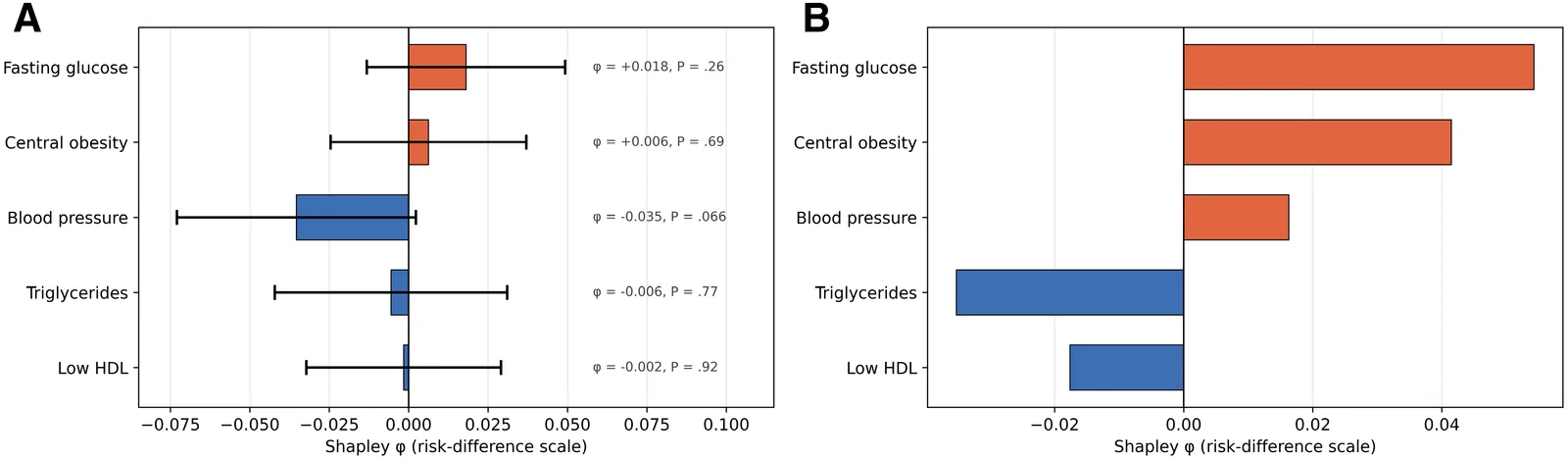
Shapley value decomposition of the DML effect estimate across the 5 MetS components. (A) Constant-effect specification with 95% CIs from the joint influence-function covariance. (B) Linear-CATE specification. All 32 coalitions estimable; Shapley weight coverage 1.00 for every component. Σφ equals *v*(N), the contrast of all 5 components present versus not all present, which is a different estimand from the ≥3 vs 0 to 2 contrast; no rescaling between them is defined. No component’s contribution differs significantly from 0. N = 1306. CATE = conditional average treatment effect, CI = confidence interval, DML = double machine learning, HDL = high-density lipoprotein, MetS = metabolic syndrome.

### 3.12. Interaction between components

No component pair showed interaction distinguishable from 0 on the additive scale after Holm adjustment (Table 9). The prespecified pair, elevated fasting glucose × central obesity, gave INT = +0.010 (95% CI, −0.115 to + 0.135; *P* = .88), an interval wide enough that it neither supports nor excludes synergy. The largest point estimate among the 9 exploratory pairs, central obesity × triglycerides at − 0.202 (−0.357 to −0.047), does not survive adjustment (Holm *P* = .11). Standard errors for these contrasts rescale each subsample influence function by N/n_*j*_; the study is not powered to resolve pairwise interaction on the additive scale.

**Table 9:** Additive-scale interaction between metabolic syndrome components, fibrosis-4 index outcome.

Pair	n both	INT	95% CI	*P*	*P* (Holm)
Fasting glucose × central obesity (prespecified primary)	710	**+0.010**	−0.115 to 0.135	.88	NA
Central obesity × triglycerides	394	−0.202	−0.357 to − 0.047	.011	.11
Blood pressure × fasting glucose	489	−0.091	−0.186 to 0.005	.062	.56
Central obesity × blood pressure	565	−0.085	−0.214 to 0.043	.19	>.99
Central obesity × low HDL	541	−0.048	−0.198 to 0.103	.53	>.99
Low HDL × blood pressure	337	+0.045	−0.055 to 0.145	.38	>.99
Triglycerides × fasting glucose	350	+0.018	−0.098 to 0.134	.76	>.99
Triglycerides × low HDL	375	−0.014	−0.148 to 0.120	.84	>.99
Low HDL × fasting glucose	433	−0.002	−0.100 to 0.095	.96	>.99
Triglycerides × blood pressure	299	+0.002	−0.109 to 0.114	.97	>.99

INT = ATE₍_1_,_1_₎ − ATE₍_1_,_0_₎ − ATE₍_0_,_1_₎; positive values indicate super-additivity. Row 1 is the prespecified primary comparison; the remaining 9 are exploratory (tier 3) with Holm adjustment. Standard errors rescale each subsample influence function by N/n_j_. NA, not applicable: the prespecified primary comparison is tested at α = 0.05 without adjustment.

Bold indicates the pre-specified primary comparison.

ATE, average treatment effect, CI = confidence interval, HDL = high-density lipoprotein, INT = additive-scale interaction contrast.

### 3.13. Sensitivity and robustness analyses

Both negative controls returned null estimates (height − 0.765, 95% CI, −2.163 to 0.634; birth quarter − 0.006, 95% CI, −0.053 to 0.041). Standing height was missing for 4 participants, so the negative-control outcome analysis used 1302 of the 1306 participants. The *E* value was 3.75 (lower bound 2.22) and Rosenbaum Γ = 3.5, both exceeding conventional thresholds. Oster δ was 0.797 under conservative assumptions (δ = 10.3 under *R*^2^max = 1.0). Evidence on unmeasured confounding is mixed (Table 10).

**Table 10:** Sensitivity analyses for unmeasured confounding, negative controls, and weighting.

Analysis	Estimate	95% CI/value	Verdict
Negative control outcome	−0.765	−2.163 to 0.634	Null ✓
Negative control exposure	−0.006	−0.053 to 0.041	Null ✓
*E* value	3.75/ 2.22	>2.0	Robust ✓
Rosenbaum Γ	3.5	>2.0	Robust ✓
Oster δ (1.3 *R*^2^)	0.797	<1.0	Not robust ✗
Oster δ (*R*^2^ = 1)	10.3	>1.0	Robust ✓
Propensity trim (0.05, 0.95)	0.145	N = 1107	Stable
Propensity trim (0.10, 0.90)	0.148	N = 1082	Stable
Without statin in the adjustment set	0.078	0.031–0.126	Sig (−49.0%)
Survey-weighted, raw	0.148	−0.013 to 0.310	Wide CI
Survey-weighted, P95-trimmed, Fay BRR	0.142	0.023–0.261	Sig
Survey-weighted, half-sample bootstrap	0.142	−0.279 to 0.563	Wide CI
Inverse-probability-of-selection weighted	0.126	0.003–0.250	Sig, attenuated
Shapley decomposition of the compound exposure	See Table 8	–	No component differs from zero ⚠

Four corrections to the previous version. The without-statin value of 0.108 was never produced by a model fit: it was the percentage change from a secondary run with numeric-coded race applied arithmetically to the primary estimate from the 1-hot specification. The reestimated value is 0.078 and the attenuation is 49.0%, not 29%. The 2 survey-weighted rows previously reported the mean across replicate weights rather than the full-sample weighted fit; the full-sample estimate and a 3rd replication method are now given. Inverse-probability-of-selection weighting is added because selection into the MASLD sample proved strongly exposure-dependent. The 2 Shapley rows are replaced by a cross-reference, because reporting point attributions without intervals invited the reading that fasting glucose and central obesity were established contributors; with intervals in hand, no component differs from zero. The negative-control outcome analysis used n = 1302, standing height being missing for 4 participants.

BRR = balanced repeated replication, CI = confidence interval, MASLD = metabolic dysfunction–associated steatotic liver disease.

#### 3.13.1. Propensity score trimming

Estimates were stable across 3 thresholds: 0.153, 0.145, and 0.148, maximum deviation 5.2%.

#### 3.13.2. Statin sensitivity

Given the extreme statin imbalance, the primary analysis was repeated, excluding statin from the confounder set. The estimate decreased from 0.153 to 0.078 (95% CI, 0.031–0.126; change 49.0%; Fig. 6). Both estimates remained significantly positive (*P* < .01). The attenuation is consistent with statin use acting as a mediator rather than a confounder: MetS triggers statin prescription, so adjusting for statin partially blocks the MetS → statin → antifibrotic pathway and leaves a direct rather than a total contrast. Under the prespecified rule of excluding mediators to preserve the total effect, the statin-excluded estimate of 0.078 is the value consistent with that rule, and 0.153 should be read as a direct-effect upper bound. Excluding statin from the adjustment set, however, risks residual confounding by unmeasured lifestyle factors correlated with statin prescription. The plausible range spans 0.078 to 0.153.

**Figure 6.**
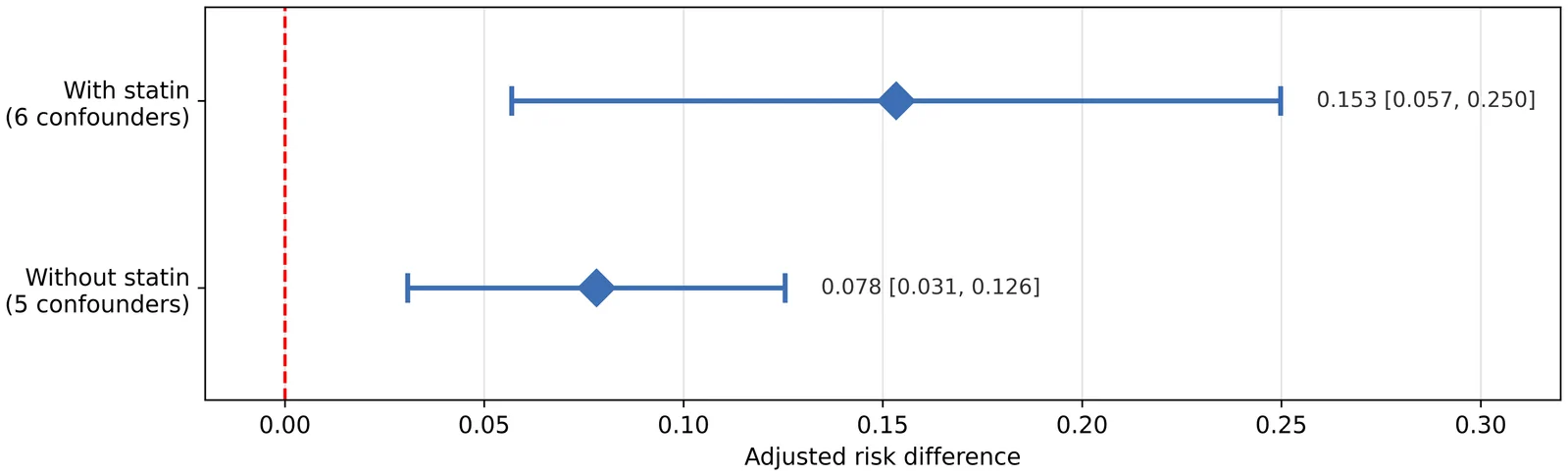
Statin sensitivity. DML with statin (6 confounders) versus without statin (5 confounders) in the adjustment set: 0.153 (0.057–0.250) versus 0.078 (0.031–0.126), a 49.0% change. Error bars: 95% CI. Both estimates remain significantly positive (*P* < .01). N = 1306. CI = confidence interval, DML = double machine learning.

#### 3.13.3. Platelet channel

Platelet counts were marginally higher, not lower, in the suprathreshold group (*P* = .66), and counts below 150 × 10^9^/L were uncommon in both groups. Because splenomegaly-mediated thrombocytopenia requires lower platelet counts in the exposed group, this mechanism cannot account for the FIB-4 result. Excluding participants with platelets below 150 × 10^9^/L gave 0.093 (95% CI, −0.019 to 0.204), and recomputing FIB-4 with platelet count held at the cohort median gave 0.084 (−0.020 to 0.189).

#### 3.13.4. MASLD eligibility

Excluding the 19 participants who met no cardiometabolic criterion gave 0.155 (95% CI, 0.064–0.246; *P* < .001), against a primary estimate of 0.153 (0.057–0.250). Restricting the sample to participants who unambiguously meet the 2023 MASLD definition therefore leaves the estimate essentially unchanged and slightly more precise. Selection into the analytic sample. Reestimating with stabilized inverse-probability-of-selection weights gave 0.126 (95% CI, 0.003–0.250; *P* = .045), an 18% attenuation with the lower confidence bound moving from 0.057 to 0.003. Weights were well-behaved (mean 1.00, standard deviation 0.24, range 0.61–2.20). This addresses selection on measured variables only.

#### 3.13.5. Survey-weighted estimation

The weighted point estimate was 0.148 with raw weights and 0.142 after trimming at the 95th percentile (weight coefficient of variation 1.13 → 0.95), against an unweighted 0.153: survey weighting did not materially change the estimate. Fay-adjusted balanced repeated replication gave 95% CIs of −0.013 to 0.310 (raw) and 0.023 to 0.261 (trimmed, *P* = .020). A conservative half-sample bootstrap returned the same point estimates with substantially wider intervals (−0.279 to 0.563, trimmed), because zeroing 1 primary sampling unit per stratum destabilizes the cross-fitted nuisance models; Fay’s adjustment avoids this by never assigning a 0o weight. Survey-weighted significance is therefore replication-scheme dependent while the point estimate is not. The unweighted sample average treatment effect remains the declared primary estimand (Fig. 7).

**Figure 7.**
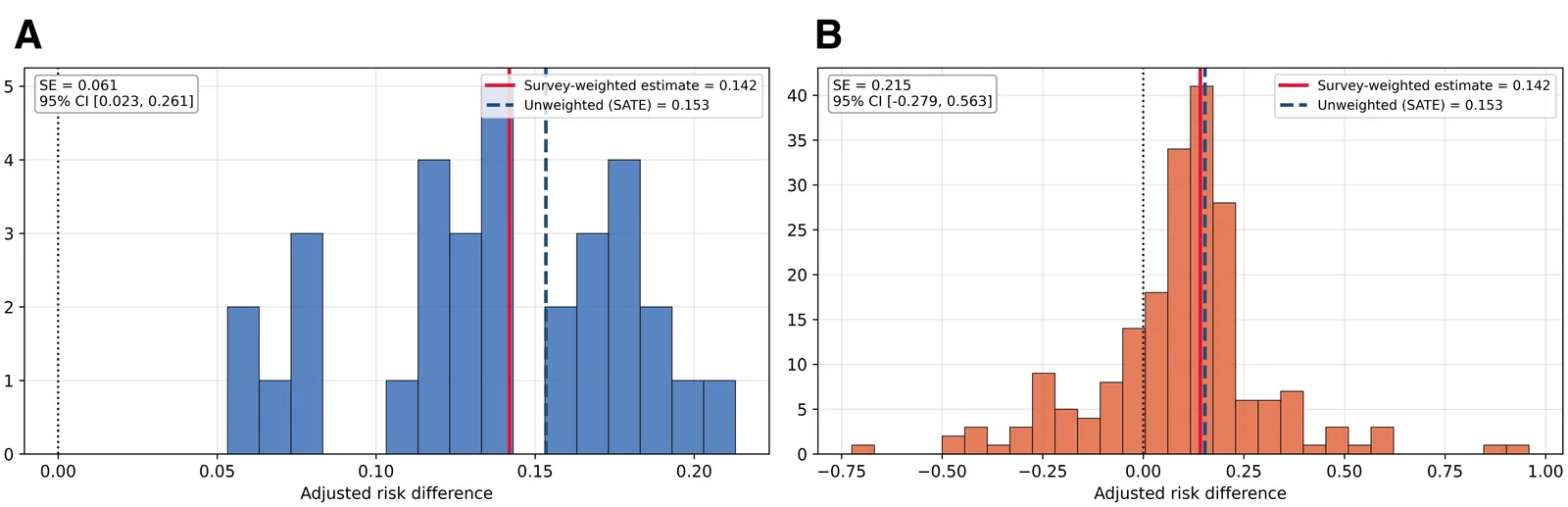
Survey-weighted replication distributions. (A) Fay-adjusted balanced repeated replication (*k* = 0.3, 32 Hadamard replicates). (B) Rao–Wu half-sample bootstrap (200 replicates). Both use P95-trimmed weights. Solid line: full-sample survey-weighted estimate (0.142); dashed line: unweighted sample average treatment effect (0.153). Replicates supply the variance only; the point estimate is not the replicate mean. N = 1306; 24 strata × 2 primary sampling units; raw weight CV 1.13, trimmed CV 0.95. CI = confidence interval, CV = coefficient of variation, SATE = sample average treatment effect, SE = standard error.

#### 3.13.6. Specification stability

Across the 10 specifications in Table 1 the estimate ranged from 0.078 to 0.163. Restricting attention to the 7 perturbations of the estimator itself, holding the sample and the adjustment set fixed, narrows the range to 0.087 to 0.163 (Fig. S1, Supplemental Digital Content 4); the 3 specifications that fall below that band are the ones that change the adjustment set or the sampling weights. The largest single source of variation was the final-stage parameterization: the primary linear-CATE specification gave 0.153 (SE 0.049), while the constant-effect specification gave 0.095 (SE 0.023; *P* < .001). Ten-fold cross-fitting gave 0.158 and 10 random seeds gave a mean of 0.158 (standard deviation 0.041, range 0.083–0.210). Substituting random forests for gradient boosting in the nuisance models gave 0.087 with an interval including 0 (−0.035 to 0.209); regularized linear and logistic nuisance models gave 0.163 (0.094–0.233). The random-forest specification is the 1 perturbation under which the finding does not remain significant.

### 3.14. External consistency

In the Tam Anh cohort (N = 106, all Vietnamese, all nondiabetic MASLD), the crude RD for FIB-4–defined fibrosis between MetS + (n = 70) and MetS − (n = 36) was 0.079, directionally concordant with the NHANES estimate. The acoustic radiation force impulse-based outcome showed a small negative difference (−0.024), not interpretable given the sample size and different elastography modality.

### 3.15. Pipeline validation

The pipeline recovered the true ATE on synthetic data (N = 5000; true 0.022) within its 95% CI (0.038; 0.012–0.065), AIPW agreed (0.013), and both negative controls were null. All 4 tests passed.

### 3.16. Summary of robustness

Across 29 robustness and validation analyses (Table 11), 19 passed, 7 required qualified interpretation, and 3 raised concerns. The dominant finding is outcome-measure sensitivity: the FIB-4 contrast of 0.153 attenuated to 0.091 after age residualization and to 0.001 for elastography at equal precision. Those that alter what FIB-4 measures cluster low; removing statin from the adjustment set gives 0.078, neutralizing the platelet term gives 0.084, removing the linear component of the age term gives 0.091, and replacing the averaged linear-CATE final stage with a constant-effect specification gives 0.095. Those that alter which participants contribute leave the estimate near the primary value; selection weighting gives 0.126 and survey weighting 0.142. The difference between the primary estimate and the conservative reading is therefore attributable to the outcome measure rather than to the sampling design.

**Table 11:** Summary of 29 robustness and validation analyses.

#	Analysis	Result	Verdict
1	DML primary (linear-CATE)	0.153, *P* = .002	✓
2	DML constant-effect specification	0.095, *P* < .001	✓
3	AIPW (numeric race)	0.132	✓
4	AIPW (indicator race)	−0.113, wide CI	⚠
5	FIB-4 age-residualized	0.091, *P* = .10	⚠
6	FibroScan	0.001, *P* = .98	✗
7	T/S/X/R Learners	0.115–0.136	✓
8	Without statin	0.078, *P* = .001	✓
9	Propensity trimming	0.145–0.148	✓
10	Survey-weighted, Fay BRR, trimmed	0.142, *P* = .020	✓
11	Survey-weighted, half-sample bootstrap	0.142, wide CI	⚠
12	Negative control outcome	Null	✓
13	Negative control exposure	Null	✓
14	Rosenbaum Γ	3.5	✓
15	*E* value lower CI	2.22	✓
16	Oster δ (1.3 *R*^2^)	0.797	✗
17	Oster δ (*R*^2^ = 1)	10.3	✓
18	BLP calibration	*P* < .001	✓
19	Shapley, interval estimates	No component ≠ 0	⚠
20	Component interaction, glucose × waist	INT = 0.010, *P* = .88	⚠
21	Age-stratified, FIB-4	*I*^2^ = 72%, absent <40	⚠
22	Age-stratified, age-residualized	*I*^2^ = 73%, same gradient; supporting evidence only	⚠
23	Age-stratified, FibroScan	*I*^2^ = 0%	✓
24	Platelet channel neutralized	0.084	✓
25	Selection weighting	0.126, *P* = .045	✓
26	Nuisance learners: random forest	0.087, CI includes 0	✗
27	Tam Anh external consistency	RD = 0.079	✓
28	Synthetic pipeline validation	4/4 pass	✓
29	MASLD eligibility restriction	0.155, *P* < .001	✓

✓ pass; ⚠ qualified; ✗ concern. Of 29 analyses, 19 passed, 7 required qualified interpretation, and 3 raised concerns. Rows 1 to 20 correspond to the 20 analyses prespecified in Protocol v2.0; rows 21 to 26 and 29 were performed at reviewer request and are post hoc; rows 27 to 28 are validation rather than robustness. Row 22 is graded as qualified because linear age residualization leaves the multiplicative age structure of FIB-4 in place, so the persistence of the gradient in the residualized outcome cannot discriminate the arithmetic of the formula from biology; the age argument rests on row 23.

AIPW = augmented inverse probability weighting, BLP = best linear predictor, BRR = balanced repeated replication, CATE = conditional average treatment effect, CI = confidence interval, DML = double machine learning, FIB-4 = fibrosis-4 index, INT = additive-scale interaction contrast, MASLD = metabolic dysfunction–associated steatotic liver disease, RD = risk difference.

## 4. Discussion

### 4.1. Principal findings

In this cross-sectional study of 1306 nondiabetic, predominantly prediabetic adults with MASLD, the suprathreshold MetS phenotype was associated with a 15.3-percentage-point higher prevalence of FIB-4–defined fibrosis risk (95% CI, 5.7–25.0; *P* = .002). The estimate attenuated to 0.091 (*P* = .10) when the linear component of FIB-4’s age dependence was removed and was essentially null for age-independent elastography (0.001; *P* = .98), whose interval excludes the FIB-4 point estimate at equal precision. The estimate therefore depends on which noninvasive test defines the outcome.

### 4.2. Comparison with existing literature

The association between MetS and hepatic fibrosis in MASLD has been consistently reported. Younossi et al reported approximately double fibrosis prevalence in NAFLD with metabolic comorbidities,^[6]^ and the 2024 European Association for the Study of the Liver Clinical Practice Guidelines identified metabolic risk factors as the strongest predictors of steatohepatitis and fibrosis progression.^[7]^ Our primary estimate, corresponding to a prevalence ratio of approximately 2.2, is directionally and quantitatively consistent.

Previous studies relied on associational methods without explicitly addressing causal identification assumptions. Our study is among the 1st to apply semiparametric causal ML methods to the MetS–fibrosis question in MASLD. Six estimators converged on 0.115 to 0.153. All 6 share LightGBM nuisance models and the same confounder set, so their agreement demonstrates robustness to the choice of effect estimator, not to nuisance specification; the latter is addressed separately in Table 1, where substituting random forests moves the estimate to 0.087 with an interval including 0.

Complementary causal evidence comes from Mendelian randomization studies using genetic instruments. Yuan et al found genetically predicted metabolic traits causally associated with NAFLD and its progression,^[39]^ supporting directionality through an entirely different identification strategy. Insulin resistance and adipose tissue inflammation are the mechanisms most often proposed for the hepatotoxicity of the MetS composite,^[40,41]^ and diabetes amplifies fibrosis risk in the same population.^[42]^

### 4.3. Interpretation of the FIB-4–FibroScan discordance

The 3 estimates disagree systematically rather than randomly: the more strongly an outcome depends on age, the larger the estimate.

#### 4.3.1. Different biology

FIB-4 combines 2 transaminases, which track hepatocellular injury and necroinflammatory activity, with platelet count, which declines progressively with fibrosis stage through reduced hepatic thrombopoietin production and, at advanced stages, splenic sequestration. It is therefore weighted toward the inflammatory end of the disease and toward advanced structural disease, with limited sensitivity to intermediate matrix deposition. Transient elastography measures tissue stiffness, which rises with cross-linked collagen but is also raised by inflammation, marked steatosis, and hepatic congestion; it is the more direct measure of matrix but not a pure 1.^[43]^ Median stiffness in this cohort was 5.1 kPa (interquartile range 4.2–6.2) and only 10.3% of participants exceeded 7.9 kPa, so appreciable matrix deposition has not yet occurred in most of them. A metabolic exposure acting on the steatohepatitis-to-fibrogenesis transition would be expected to move transaminases before it moves stiffness, and the observed pattern is consistent with that sequence. That sequence does not by itself predict, however, the age gradient that the FIB-4 outcomes show and elastography does not.

#### 4.3.2. Formula-driven inflation

The groups differ in mean age by 11.4 years, and age enters FIB-4 multiplicatively (*r* = 0.468). Age residualization reduced the estimate to 0.091 but is a partial correction because residualizing on a linear age term cannot remove a multiplicative dependence. The age-stratified analysis is the more informative test, and its weight falls on the outcome that contains no age term: elastography showed no heterogeneity across strata (*I*^2^ = 0%), whereas both FIB-4 outcomes did (*I*^2^ = 72% and 73%). The gradient is therefore specific to FIB-4, and its age term is the most economical explanation. That the gradient survives linear residualization is consistent with this reading rather than evidence against it, because the residualized score retains the multiplicative age structure; we treat that result as supporting evidence only.

#### 4.3.3. Reverse causation through the platelet term

Advancing hepatic disease lowers platelet count through splenic sequestration, raising FIB-4 mechanically while leaving stiffness unaffected in the same direction. This pathway requires clinically significant portal hypertension, and therefore advanced fibrosis or cirrhosis, which is rare in a cohort with a median stiffness of 5.1 kPa. The mechanism predicts lower platelet counts in the suprathreshold group. Counts were marginally higher, so it cannot account for the finding. Neutralizing the platelet channel nevertheless attenuates the estimate to 0.084, placing the platelet term alongside the age term as a component of FIB-4 that inflates the estimate relative to the underlying biology.

FIB-4 carries an age term and a platelet term. We therefore interpret the study through the elastography estimate, with the age-residualized estimate as supporting evidence, carrying the conservative estimate forward and treating the primary FIB-4 result as the upper end of the 0.08 to 0.10 band on which perturbations of what FIB-4 measures converge, with the elastography estimate as the conservative bound.

### 4.4. Interpretability of the causal ML pipeline

ML causal estimators are often described as opaque. We therefore state what is and is not inspectable in this pipeline. Assessed against the 3 dimensions proposed by Mediouni et al,^[29]^ this pipeline is interpretable in a particular and limited sense. On individual input features, the Shapley decomposition operates on the effect estimate rather than on model predictions, so the attributions are on the RD scale and inherit the orthogonality of the underlying fits; they decompose a compound exposure but do not identify mediation, and with interval estimates in hand, none of the 5 components is distinguishable from 0. On traceability, the quantity reported is the sample mean of an orthogonal score, and the gradient-boosted learners enter only through 2 nuisance functions to which the estimate is 1st-order insensitive, a stronger transparency property than that of any single-stage prediction model, though it does not make the nuisance models themselves inspectable, and Table 1 shows that the choice of nuisance learner family moves the estimate from 0.087 to 0.163. On interactions, the factorial contrasts test additivity between component pairs directly, and the prespecified pair proved additive. We can trace and decompose the effect estimate, but the nuisance models themselves remain uninspectable. Orthogonality limits the resulting bias without eliminating it.

### 4.5. Strengths

First, this is among the 1st applications of DML to a hepatology question in a nationally representative dataset, with stated causal identification assumptions, Neyman-orthogonal moment conditions, and cross-fitted nuisance models. Second, the sensitivity analysis suite (20 analyses prespecified in the protocol and 9 added at reviewer request) exceeds the typical standard for observational liver disease studies and includes analyses that could have overturned the finding: age stratification, platelet neutralization, selection weighting, and nuisance learner substitution. Third, unfavorable results (the elastography null, the age gradient specific to FIB-4, AIPW instability, Oster δ < 1, and the random-forest specification whose interval includes 0) are reported in full. Fourth, the Shapley decomposition is reported with interval estimates that show the component ranking to be descriptive only. Fifth, external consistency with the Tam Anh cohort provides preliminary cross-ethnic comparison.

### 4.6. Limitations

The estimand is structurally constrained in 3 ways that adjustment cannot repair. Metabolic syndrome is a threshold on a composite lying on the same physiological axis as the disease state that defines the population, so the contrast is between severity strata. The ≥3/5 threshold makes the exposure compound, with 16 realizing configurations, so consistency holds only in an averaged sense. And restriction to MASLD conditions on a collider, since all 5 harmonized components are themselves qualifying MASLD criteria. Only the 3rd is quantifiable: exposed participants were a 3rd more likely to enter the analytic sample than unexposed ones (40.7% vs 30.7%; *P* < .001), principally through central obesity, and weighting for the measured part attenuated the estimate by 18% and moved the lower bound to 0.003. Selection through unmeasured determinants of hepatic steatosis is not correctable by weighting and remains the principal unresolved threat.

Two features of measurement bound the interpretation. The design is cross-sectional, so temporality is assumed rather than observed; MetS typically antedates fibrosis in the natural history of MASLD,^[44]^ but DML addresses confounding and contributes nothing to ordering. FIB-4 also incorporates age mechanically. Linear age residualization removes only the linear component of that age dependence, so the persistence of the stratum gradient in the residualized outcome does not show that the gradient is independent of age; the absence of heterogeneity for age-independent elastography (*I*^2^ = 0%) indicates that the gradient is specific to FIB-4 and most likely arises from its age term. What these data cannot separate is the age term of the formula from age-related biology in the constituents of the score, and that separation requires an outcome anchored to histology. The discordance with elastography, estimated with the same SE (0.001; 95% CI, −0.094 to 0.097), is the measured expression of that limit.

The estimate is specification-dependent to a degree a single number would conceal. The final-stage parameterization alone moves the point estimate by about 40%, and substituting random forests for gradient boosting in the nuisance models yields an interval including 0; the range across 10 specifications, 0.078 to 0.163, is the appropriate summary of what these data support. Evidence on unobserved confounding is mixed: an *E* value of 3.75 and a Rosenbaum Γ of 3.5 would require implausibly strong confounders, but Oster’s δ of 0.797 falls below the conventional threshold, and unmeasured PNPLA3 and TM6SF2 genotype,^[45]^ physical activity, and diet may contribute.

Finally, 73.4% of the sample was prediabetic. These estimates describe metabolically unhealthy individuals with MASLD who have not yet reached the diagnostic threshold for diabetes. They say least about the lean and metabolically healthy phenotypes, in which the drivers of fibrosis may be entirely different, the relationship is least understood, and the subthreshold comparison group would be least confounded by prevalent dysglycemia.

### 4.7. Clinical implications

If a causal interpretation is supported by future longitudinal studies, 3 implications follow for the predominantly prediabetic MASLD population studied here. First, fasting glucose and central obesity ranked highest among the components, but their CIs include 0, so these findings motivate rather than justify component-specific targeting. Semaglutide improved histological resolution of steatohepatitis without significant fibrosis improvement in a phase 2 trial,^[46]^ and subsequently improved both steatohepatitis resolution and fibrosis in the phase 3 ESSENCE trial;^[47]^ resmetirom has demonstrated both steatohepatitis resolution and fibrosis improvement in established metabolic dysfunction–associated steatohepatitis with *F*2–*F*3 fibrosis.^[48]^ Whether MetS-targeted interventions prevent fibrosis onset, as opposed to treating established disease, remains unproven.

Second, the FIB-4 contrast was concentrated above age 40 and absent below it, whereas the elastography contrast showed no age pattern. The null below 40 is precisely estimated, but because the gradient appears to be specific to FIB-4 it says more about the marker than about fibrosis, and it should not guide age-targeted screening until it has been tested against an outcome that does not contain age. Third, the discordance between FIB-4 and elastography argues for using more than 1 noninvasive test when fibrosis is the outcome of a causal analysis.

## 5. Conclusions

In this cross-sectional analysis of 1306 nondiabetic, predominantly prediabetic adults with MASLD, the suprathreshold MetS phenotype was associated with a higher prevalence of FIB-4–defined elevated fibrosis risk (adjusted RD 0.153; 95% CI, 0.057–0.250; *P* = .002), but the association attenuated to 0.091 (*P* = .10) when the linear component of the age dependence of FIB-4 was removed and was 0.001 (*P* = .98) for age-independent transient elastography, whose CI excludes the FIB-4 point estimate at equal precision.

Perturbations that alter what FIB-4 measures converge on 0.08 to 0.10, while perturbations that alter sample composition leave the estimate near 0.13 to 0.15, indicating that the outcome measure rather than the sampling design accounts for the difference. Fasting glucose and central obesity ranked highest in a Shapley decomposition, but no component’s contribution differed significantly from 0, and no component pair showed additive-scale interaction after adjustment.

Three design features bound what these numbers support. The exposure is a threshold on a composite lying on the same physiological axis as the disease state, so the estimate is a contrast between severity strata rather than an interventional effect; selection into the study was itself strongly exposure-dependent. The cross-sectional design cannot establish that MetS precedes fibrosis, and DML addresses confounding but contributes nothing to temporal ordering. And the estimate depends materially on which noninvasive test defines the outcome.

These findings therefore generate hypotheses for confirmatory testing rather than providing definitive causal evidence. They apply to the predominantly prediabetic MASLD population studied here, in which 73.4% of participants had prediabetes, and cannot be extrapolated to metabolically healthy or lean MASLD, in whom the drivers of fibrosis may be entirely different. The hypotheses are specific and testable: that fasting glucose and central obesity carry the fibrogenic weight within the composite; that the FIB-4–elastography discordance arises from the age term of FIB-4 rather than from an early steatohepatitis-to-fibrogenesis transition, the reading favored by the age-stratified elastography estimates but not settled by them; and that resolution of the suprathreshold phenotype reduces fibrosis progression. Longitudinal cohorts with biopsy-confirmed endpoints, age-stratified by design, are required to test them.

## Acknowledgments

The authors thank the participants and staff of NHANES and Tam Anh General Hospital for their contribution to this research. No individual is named in this Acknowledgments. This research received no specific grant from any funding agency, and the authors declare no financial or nonfinancial interests that could be perceived as a conflict of interest.

## Author contributions

**Conceptualization:** Hien-Huu Tran, Lua Thi Ngo.

**Data curation:** Van Trung Huynh, Hien-Huu Tran.

**Formal analysis:** Hien-Huu Tran, Lua Thi Ngo.

**Investigation:** Van Trung Huynh, Hien-Huu Tran.

**Methodology:** Hien-Huu Tran, Lua Thi Ngo.

**Project administration:** Lua Thi Ngo, Hien-Huu Tran.

**Resources:** Van Trung Huynh.

**Software:** Hien-Huu Tran.

**Supervision:** Lua Thi Ngo.

**Validation:** Van Trung Huynh, Lua Thi Ngo.

**Visualization:** Hien-Huu Tran.

**Writing – original draft:** Hien-Huu Tran.

**Writing – review & editing:** Van Trung Huynh, Lua Thi Ngo, Hien-Huu Tran.








