# Supplementary material for: Metabolic syndrome and liver fibrosis in nondiabetic MASLD: A cross-sectional double machine learning study
Source: Medicine (Baltimore). 2026 Nov 6;105(45):e51316. doi: 10.1097/MD.0000000000051316 (PMC13747392; doi:10.1097/MD.0000000000051316)
Supplement: Supplementary file 1 [file medi-105-e51316-s001.docx]

## **Supplementary Table S1. Frequency of all 32 metabolic syndrome component configurations (N = 1,306)**

Every one of the 32 possible configurations was observed, so the compound exposure is realized across its full configuration space. Waist = central obesity; TG = elevated triglycerides; HDL = low HDL cholesterol; BP = elevated blood pressure; FPG = elevated fasting plasma glucose. k = number of components present.

| **Phenotype** | **k** | **Components present** | **n** | **FIB-4 ≥1.3, n (%)** |
| --- | --- | --- | --- | --- |
| Supra-threshold (MetS+) | 5 | Waist + TG + HDL + BP + FPG | 163 | 68 (41.7) |
| Supra-threshold (MetS+) | 4 | Waist + TG + HDL + FPG | 73 | 30 (41.1) |
| Supra-threshold (MetS+) | 4 | Waist + HDL + BP + FPG | 64 | 17 (26.6) |
| Supra-threshold (MetS+) | 4 | Waist + TG + HDL + BP | 47 | 14 (29.8) |
| Supra-threshold (MetS+) | 4 | Waist + TG + BP + FPG | 32 | 18 (56.2) |
| Supra-threshold (MetS+) | 4 | TG + HDL + BP + FPG | 30 | 16 (53.3) |
| Supra-threshold (MetS+) | 3 | Waist + BP + FPG | 154 | 55 (35.7) |
| Supra-threshold (MetS+) | 3 | Waist + HDL + FPG | 73 | 8 (11.0) |
| Supra-threshold (MetS+) | 3 | Waist + TG + HDL | 38 | 7 (18.4) |
| Supra-threshold (MetS+) | 3 | Waist + TG + FPG | 21 | 6 (28.6) |
| Supra-threshold (MetS+) | 3 | Waist + HDL + BP | 19 | 5 (26.3) |
| Supra-threshold (MetS+) | 3 | TG + HDL + FPG | 16 | 3 (18.8) |
| Supra-threshold (MetS+) | 3 | Waist + TG + BP | 10 | 4 (40.0) |
| Supra-threshold (MetS+) | 3 | TG + BP + FPG | 9 | 5 (55.6) |
| Supra-threshold (MetS+) | 3 | HDL + BP + FPG | 6 | 1 (16.7) |
| Supra-threshold (MetS+) | 3 | TG + HDL + BP | 5 | 2 (40.0) |
| Sub-threshold (MetS−) | 2 | Waist + FPG | 130 | 27 (20.8) |
| Sub-threshold (MetS−) | 2 | Waist + BP | 76 | 14 (18.4) |
| Sub-threshold (MetS−) | 2 | Waist + HDL | 64 | 2 (3.1) |
| Sub-threshold (MetS−) | 2 | BP + FPG | 31 | 5 (16.1) |
| Sub-threshold (MetS−) | 2 | Waist + TG | 10 | 0 (0.0) |
| Sub-threshold (MetS−) | 2 | HDL + FPG | 8 | 1 (12.5) |
| Sub-threshold (MetS−) | 2 | TG + FPG | 6 | 1 (16.7) |
| Sub-threshold (MetS−) | 2 | HDL + BP | 3 | 0 (0.0) |
| Sub-threshold (MetS−) | 2 | TG + BP | 3 | 0 (0.0) |
| Sub-threshold (MetS−) | 2 | TG + HDL | 3 | 0 (0.0) |
| Sub-threshold (MetS−) | 1 | Waist | 100 | 9 (9.0) |
| Sub-threshold (MetS−) | 1 | FPG | 41 | 5 (12.2) |
| Sub-threshold (MetS−) | 1 | BP | 14 | 3 (21.4) |
| Sub-threshold (MetS−) | 1 | TG | 9 | 2 (22.2) |
| Sub-threshold (MetS−) | 1 | HDL | 3 | 0 (0.0) |
| Sub-threshold (MetS−) | 0 | none | 45 | 3 (6.7) |

*The supra-threshold phenotype (k ≥ 3) is realized by 16 distinct configurations and the sub-threshold phenotype (k ≤ 2) by a further 16. The primary estimate is a weighted average over this configuration mix and will not transport unchanged to a population with a different mix.*
