# Supplementary material for: Metabolic syndrome and liver fibrosis in nondiabetic MASLD: A cross-sectional double machine learning study
Source: Medicine (Baltimore). 2026 Nov 6;105(45):e51316. doi: 10.1097/MD.0000000000051316 (PMC13747392; doi:10.1097/MD.0000000000051316)
Supplement: Supplementary file 2 [file medi-105-e51316-s003.docx]

## **Supplementary Table S2. Age-stratified estimates under the constant-effect specification (N = 1,306)**

Companion to Table 11, which reports the same strata under the primary linear-CATE specification. Here the confounders enter as controls rather than as effect modifiers, giving the canonical partially linear estimand.

| **Outcome** | **Age stratum** | **N** | **Events** | **Adjusted RD** | **95% CI** | ***P*** |
| --- | --- | --- | --- | --- | --- | --- |
| FIB-4 ≥1.3 | <40 | 426 | 25 | 0.031 | −0.018 to 0.080 | .21 |
|  | 40–59 | 462 | 135 | 0.146 | 0.071–0.222 | <.001 |
|  | ≥60 | 418 | 171 | 0.123 | 0.008–0.239 | .037 |
| FIB-4 age-residualized | <40 | 426 | 79 | 0.035 | −0.036 to 0.106 | .33 |
|  | 40–59 | 462 | 146 | 0.179 | 0.099–0.259 | <.001 |
|  | ≥60 | 418 | 106 | 0.140 | 0.039–0.240 | .007 |
| FibroScan ≥7.9 kPa | <40 | 426 | 39 | 0.026 | −0.033 to 0.086 | .39 |
|  | 40–59 | 462 | 46 | 0.053 | −0.002 to 0.109 | .061 |
|  | ≥60 | 418 | 50 | 0.051 | −0.022 to 0.123 | .17 |

*Adjusted risk difference from LinearDML with confounders passed as controls, LightGBM nuisance models (400 trees), five-fold cross-fitting, seed 42, race one-hot encoded. Under this specification both older strata reach significance for the FIB-4 outcome and for the age-residualized outcome, whereas the age-independent elastography outcome does not reach significance in any stratum. The stratum gradient therefore persists after the linear component of the age dependence has been removed from the outcome. Because age enters FIB-4 multiplicatively, this residualization is partial and the persistence of the gradient cannot discriminate the arithmetic of the formula from biology; the comparison that bears on the age question is with the elastography outcome, which contains no age term and shows no heterogeneity across strata (main text, Results).*

### **Minimum detectable effect by stratum, FIB-4 outcome**

| **Age stratum** | **N** | **Events** | **Linear-CATE SE** | **MDE (80%)** | **Constant-effect SE** | **MDE (80%)** |
| --- | --- | --- | --- | --- | --- | --- |
| <40 | 426 | 25 | 0.0252 | 0.071 | 0.0250 | 0.070 |
| 40–59 | 462 | 135 | 0.0897 | 0.251 | 0.0387 | 0.108 |
| ≥60 | 418 | 171 | 0.1321 | 0.370 | 0.0589 | 0.165 |

*Minimum detectable effect at 80% power and two-sided α = 0.05. Under the primary linear-CATE specification only the youngest stratum is adequately powered; under the constant-effect specification all three are.*
